# A widespread role of the motif environment in transcription factor binding across diverse protein families

**DOI:** 10.1101/gr.184671.114

**Published:** 2015-09

**Authors:** Iris Dror, Tamar Golan, Carmit Levy, Remo Rohs, Yael Mandel-Gutfreund

**Affiliations:** 1 Faculty of Biology, Technion–Israel Institute of Technology, Technion City, Haifa 32000, Israel;; 2 Molecular and Computational Biology Program, Departments of Biological Sciences, Chemistry, Physics, and Computer Science, University of Southern California, Los Angeles, California 90089, USA;; 3 Department of Human Genetics and Biochemistry, Sackler Faculty of Medicine, Tel Aviv University, Tel Aviv 69978, Israel

## Abstract

Transcriptional regulation requires the binding of transcription factors (TFs) to short sequence-specific DNA motifs, usually located at the gene regulatory regions. Interestingly, based on a vast amount of data accumulated from genomic assays, it has been shown that only a small fraction of all potential binding sites containing the consensus motif of a given TF actually bind the protein. Recent in vitro binding assays, which exclude the effects of the cellular environment, also demonstrate selective TF binding. An intriguing conjecture is that the surroundings of cognate binding sites have unique characteristics that distinguish them from other sequences containing a similar motif that are not bound by the TF. To test this hypothesis, we conducted a comprehensive analysis of the sequence and DNA shape features surrounding the core-binding sites of 239 and 56 TFs extracted from in vitro HT-SELEX binding assays and in vivo ChIP-seq data, respectively. Comparing the nucleotide content of the regions around the TF-bound sites to the counterpart unbound regions containing the same consensus motifs revealed significant differences that extend far beyond the core-binding site. Specifically, the environment of the bound motifs demonstrated unique sequence compositions, DNA shape features, and overall high similarity to the core-binding motif. Notably, the regions around the binding sites of TFs that belong to the same TF families exhibited similar features, with high agreement between the in vitro and in vivo data sets. We propose that these unique features assist in guiding TFs to their cognate binding sites.

Transcriptional regulation is highly dependent on the binding of transcription factors (TFs) to short DNA binding motifs (Matys et al. 2003; Bryne et al. 2008). Whereas such short sequence motifs can appear a myriad of times in the genome, only a small fraction is bound by the corresponding TF (Ren et al. 2000; Iyer et al. 2001; Harbison et al. 2004). Moreover, recent ENCODE data suggest that, on average, 99.8% of putative binding motifs in the genome are not bound by the respective TF (Wang et al. 2012). It is therefore clear that the presence of a binding motif per se is not sufficient for TF binding.

An important question that arises from these findings is what distinguishes a region containing the motif that is bound by the TF from a region containing a similar motif that is not bound in a specific cell type at a given time point. Over the past few decades, many studies have addressed this question (for review, see Slattery et al. 2014). One widely accepted approach suggests that an interplay exists between TF binding and chromatin accessibility (Thurman et al. 2012; Barozzi et al. 2014). Specifically, it has been suggested that many TFs preferentially bind in regions of open chromatin (Song et al. 2011), by actively opening condensed chromatin, joining chromatin modifying factors, or binding to constitutively opened chromatin. Combinatorial interactions of TFs are also believed to facilitate binding (Lelli et al. 2012). According to this view, the recognition of functional binding sites by a TF is dictated not only by the core-binding motif but also by a combination of adjacent motifs (Slattery et al. 2011; Martinez and Rao 2012; Yanez-Cuna et al. 2012; Kazemian et al. 2013; Crocker et al. 2015).

Clearly, chromatin accessibility and combinatorial binding play an important role in directing TFs to functional regions in vivo. However, selective binding of motifs by TFs has also been observed in a variety of in vitro experiments (Noyes et al. 2008; Badis et al. 2009; Berger and Bulyk 2009; Zhao et al. 2009; Slattery et al. 2011; Enuameh et al. 2013; Gordân et al. 2013; Jolma et al. 2013; Afek et al. 2014; Weirauch et al. 2014; Abe et al. 2015; Levo et al. 2015). These in vitro studies show that TFs can bind to different sequences containing a similar motif with a large range of different affinities, which suggests that TF-DNA binding specificity is influenced by the DNA context surrounding the motif. Indeed, the contribution of the regions directly flanking the motif to binding specificity in vitro has been demonstrated for a small number of TFs (Gordân et al. 2013; Afek et al. 2014; Yang et al. 2014; Levo et al. 2015). The sequence environment of a motif has also been shown to contribute to transcriptional regulation by the TF Cone-rod homeobox (CRX) (White et al. 2013).

Here, we aimed at performing a large-scale analysis investigating the inherent contribution of the extended regions surrounding hundreds of TF binding motifs. This analysis revealed significant differences in the nucleotide content between bound and unbound regions extending far beyond the consensus motif, showing high agreement between the in vivo and in vitro data. Notably, TFs belonging to the same protein families demonstrated similar sequence preferences in the extended regions around the binding motifs. Moreover, we show that the preferred nucleotide content has an overall high similarity to the core motif and exhibits unique DNA structural features. These results emphasize the intrinsic role of the sequence environment in protein–DNA recognition. We propose that the sequence environment around the consensus motif may help in guiding the TFs to their cognate binding sites.

## Results

### Sequence compositions surrounding TF binding motifs contribute to in vitro binding preferences

**Figure 1.**
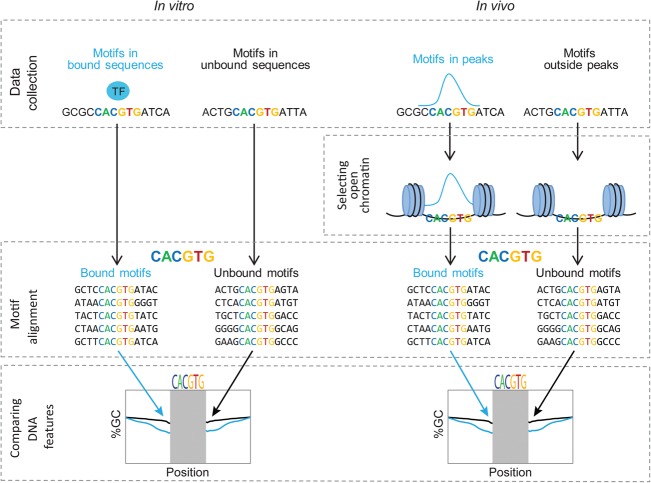
A flowchart describing the approach used for finding preferences in regions surrounding TF binding motifs. (*Left*) For each TF, a pool of bound and unbound sequences was collected from HT-SELEX data of human and mouse TFs (Jolma et al. 2013). Both sequence pools were filtered, keeping only sequences possessing the published TF binding motif. The sequences were further aligned relative to the TF binding motif. Nucleotide content of the sequences flanking the binding motif was compared between the bound and unbound groups. (*Right*) For each TF, a pool of bound and unbound sequences was collected from ChIP-seq data (Yan et al. 2013). Both pools were filtered, keeping only sequences possessing the TF binding motif in open chromatin. The sequences were further aligned relative to the motif. Finally, the nucleotide content of the sequences surrounding the binding motif in the bound and unbound groups was compared between the two groups.

**Figure 2.**
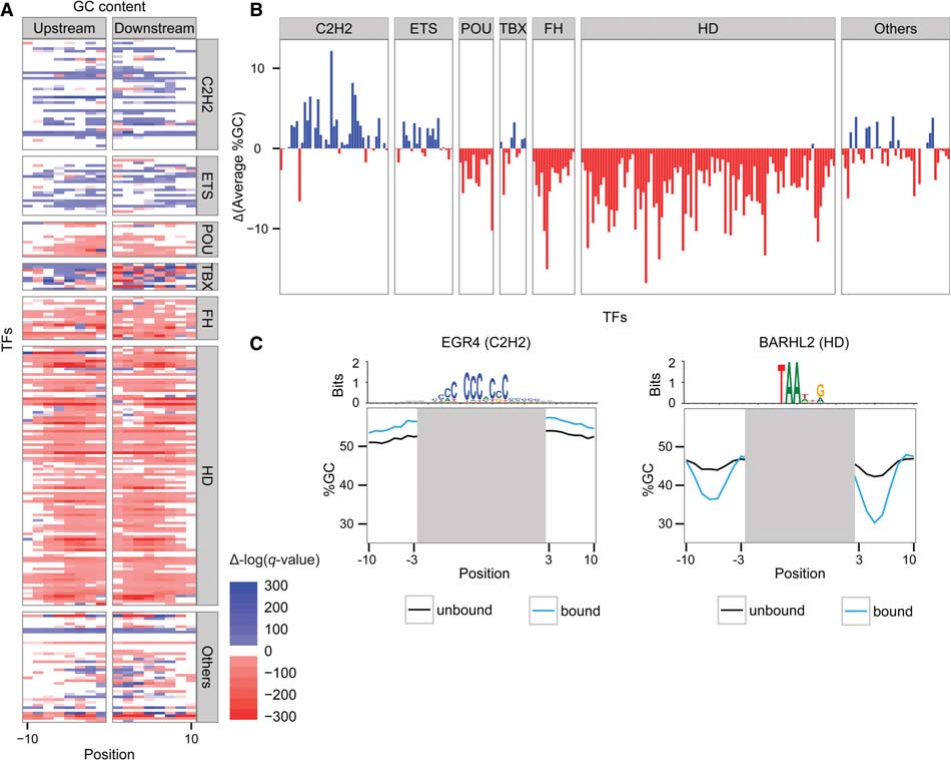
Differences in nucleotide content of the regions surrounding TF motifs in bound and unbound sequences extracted from in vitro data. (*A*) Heat map representing the differences in GC content 10 bp upstream of and downstream from the core motifs: Red indicates positions at which the GC content was significantly lower in the bound motifs; blue, positions at which the GC content was significantly higher in the bound compared with the unbound motifs (the color intensity represents the statistical significance). The TFs were grouped by the different TF families (FH, forkhead; HD, homeodomain). The positions correspond to the core-binding motif. (*B*) Differences between the average GC content (%GC) of the bound and the unbound sequences: Red indicates TFs that prefer binding in regions with high AT content; blue, TFs that prefer binding to regions with high GC content. (*C*) The GC content (%GC) in each position of the bound (blue) and unbound (black) sequences for two TFs: the EGR4 TF (C2H2 zinc finger; *left*), and the BARHL2 TF (homeodomain; *right*). Motif logos representing the aligned bound sequences are shown *above*.

In order to study intrinsic binding preferences, we first concentrated on in vitro binding assays and analyzed HT-SELEX data for 239 TFs (Supplemental Table 1; Jolma et al. 2013). Specifically, we were interested in comparing the sequence composition in regions surrounding the motifs found in bound versus unbound sequences. To this end, we collected a set of bound sequences for each TF and a set of unbound sequences. We further filtered both sets based on the existence of a previously published binding motif of each TF (Jolma et al. 2013) and aligned the sequences accordingly. A flowchart representing our analysis is shown in Figure 1. This process resulted in two distinct sets of sequences, one for bound and a second for unbound sites, which share the known TF binding motif. This allowed us to concentrate on differences in the motif environment, which presumably contributes to the differential binding status of the sequences in each of the groups. We first examined the differences in sequence composition surrounding bound versus unbound motifs by comparing the GC content in each position of the aligned sequences, 10 bp upstream of and downstream from the core motif excluding positions of the core motif and 2 bp upstream of and downstream from the core motif, and evaluated the statistical significance of the differences (see Methods). By using this approach, we found that the majority of TFs show differences in the GC composition surrounding their binding motifs (Supplemental Figs. 1, 2, 3A; for a comparison of each nucleotide separately, see Supplemental Fig. 4A), with a difference of up to 16% in GC content between the bound and unbound sequences (3.4% on average) (Fig. 2B). On average, 60% of the positions surrounding each TF binding motif had significant differences in their GC content between the bound and unbound pools (*q*-value ≤ 0.05). For comparison, when randomly shuffling the labels between the bound and unbound sequences, no significant differences were detected (Supplemental Fig. 5). Specifically, we found that 138 TFs preferred binding to motifs surrounded by high AT content, while 49 TFs preferred binding to motifs located in regions of high GC content (Fig. 2A). When we clustered TFs based on their Pfam binding domain (Finn et al. 2014), we found that TFs belonging to evolutionary related domains often have similar environmental preferences (Fig. 2A). For example, we found that most of the TFs belonging to the homeodomain family (88 out of 96 members), the POU family (10 out of 13), and the forkhead family (14 out of 16) prefer binding to regions with low GC content surrounding the core motif, as opposed to C2H2 zinc finger (19 out of 41) and ETS TFs (12 out of 22), which demonstrated a preference for binding to GC-rich regions. In Figure 2C, we illustrate the GC preference of EGR4, a C2H2 TF. As shown in the figure, EGR4 prefers binding to sequences that are enriched in GC. The opposite behavior was observed for BARHL2, a homeodomain TF, which prefers binding to motifs residing in regions characterized by lower GC content. To ensure that the differences are not the result of experimental or statistical biases, we conducted several control tests, all confirming that TFs from different families have characteristic sequence preferences in the environment surrounding bound motifs (see Supplemental Material).

### Sequence preferences detected in regions surrounding in vivo binding sites

The environmental preference observed in the HT-SELEX data suggests that TF binding is influenced by additional information beyond the core motif. Next, we asked whether these intrinsic preferences are also found in vivo. To this end, we analyzed ChIP-seq data for 56 TFs (Supplemental Table 2; Yan et al. 2013). As described in Figure 1, we searched the genome for appearances of the TF binding motifs and divided them into two groups: motifs found inside ChIP-seq peaks (bound motifs) and those falling outside ChIP-seq peaks (unbound motifs).

As aforementioned, it is well established that TF binding sites are located preferentially in regions of accessible chromatin (Wang et al. 2012). Moreover, regions of open chromatin possess high GC content compared with the rest of the genome (Fenouil et al. 2012). Thus, when comparing sequences that are either bound or unbound by the TF, we might find differences that reflect the differential GC content in open and closed chromatin rather than the intrinsic preference of the TF. To overcome this genomic bias, we incorporated DNase I hypersensitivity data (see Methods) in order to separate the genomic sequences into open and closed regions, thus enabling us to compare bound and unbound motifs in similar environments. Next, analogous to the in vitro analysis, we compared the nucleotide content at each position surrounding the core motifs, examining 300 bp upstream of and downstream from the motif.

**Figure 3.**
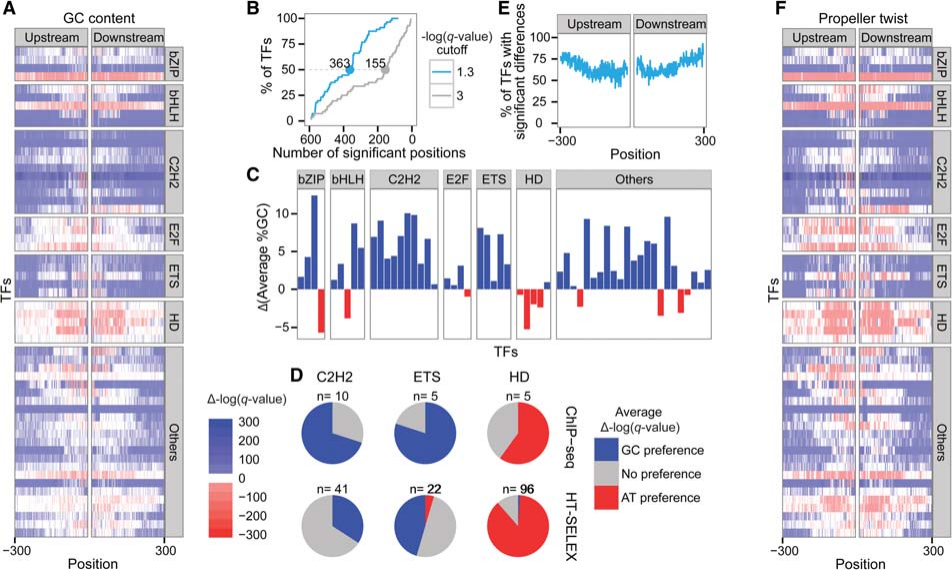
Differences in features of the regions surrounding TF motifs in bound and unbound sequences extracted from in vivo data. (*A*) Heat map representing the differences in GC content 300 bp upstream of and downstream from the core motifs: Red indicates positions at which the GC content was significantly lower in the bound motifs; blue, positions at which the GC content was significantly higher in the bound compared with the unbound motifs (the color intensity represents the significance). The TFs were grouped by the different TF families (HD for homeodomain). The positions correspond to the core-binding motif. (*B*) Cumulative plot representing the proportion of TFs as a function of the number of surrounding positions that differ significantly between the bound and unbound groups using two different thresholds to define significant differences: −log(*q*-value) ≥ 1.3 in blue and ≥3 in gray. (*C*) Differences between the average %GC of the bound and the unbound sequences: Red indicates TFs that prefer binding to regions with high AT content; blue, TFs that prefer binding to regions with high GC content. (*D*) Pie charts showing the number of TFs with significant nucleotide content differences for the three TF families shared between the in vitro (*bottom*) and in vivo (*top*) data. Blue represents GC preferences [Δ−log(*q*-value) ≥ 1.3]; gray, no significant preferences; and red, AT preferences [Δ−log(*q*-value) ≤ −1.3]. (*E*) Plot showing the percentage of TFs with significant nucleotide content differences [−log(*q*-value) ≥ 1.3] for each position 300 bp upstream of and downstream from the core motif. (*F*) Heat map representing the differences in propeller twist 300 bp upstream of and downstream from the core motifs: Red indicates positions at which the propeller twist was lower in the bound motifs; blue, positions at which the propeller twist was less pronounced in the bound compared with the unbound motifs (the color intensity represents the statistical significance). The TFs are grouped by the different TF families (HD for homeodomain). The positions correspond to the core-binding motif.

Consequently, when comparing the sequences surrounding bound and unbound motifs, concentrating on open chromatin, we found significant differences in GC content (Fig. 3A; Supplemental Figs. 3B, 4B, 6), with an overall good agreement with the in vitro GC preferences (Supplemental Fig. 7). Specifically, we noticed that in half of the TFs, >50% of the 300 nucleotides (nt) demonstrated significant differences in their GC content (Fig. 3B). Differences of up to 12% in GC content were observed (for CREB3L4), with an average of 4.3% over all TFs (Fig. 3C). Consistent with the in vitro results, we found that in vivo, TFs sharing homologous DNA binding domains (as defined by Pfam) (Finn et al. 2014) often share the same GC preferences. We noticed that similar to the in vitro results, TFs from the homeodomain family tend to bind motifs embedded within higher AT content regions, whereas members of the C2H2 and ETS families seem to bind preferentially to regions of higher GC content (Fig. 3D). When examining the distribution of the significant positions relative to the binding motif, we noticed that differences in GC content extend far beyond the core motif, reaching 300 bp upstream of and downstream from the core motif (Fig. 3E). It has been previously shown that the promoters of different gene classes possess an overall higher or lower than average GC content (Smith et al. 2005). In order to control for GC biases, which could result from such regulatory regions, we removed all TF peaks that are found within promoters having either high or low GC content (top and bottom 10%, respectively) and repeated the analysis and found overall similar results (Supplemental Fig. 8).

Taken together, we found that most TFs demonstrate strong preference to bind within regions possessing specific nucleotide content. Moreover, we found that the TF preferences for specific sequence environments were similar in the in vitro and in vivo binding assays, proposing that these preferences demonstrate an inherent binding property of the TFs.

### TF binding sites are preferentially found in homotypic environments

**Figure 4.**
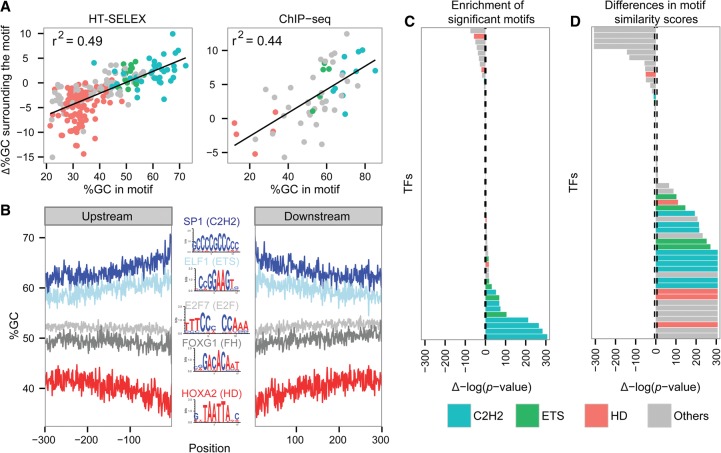
Differences in motif similarities. (*A*) Correlation between %GC of the bound motifs and Δ%GC surrounding bound compared with unbound motifs, in vitro (*left*) and in vivo (*right*); black line shows the linear regression trend line. The *r*^2^ of the trend line is shown. The TFs are colored according to the color code used for TF families: cyan for C2H2 TFs, green for ETS TFs, red for homedomains, and all others in gray. (*B*) %GC upstream of and downstream from motifs found in sequences bound by SP1 (dark blue), ELF1 (light blue), E2F7 (light gray), FOXG1 (dark gray), and HOXA2 (red). Logos of the TF-bound motifs are shown in the *center*: A and T bases are colored in red; G and C bases, in blue. (*C*) Wilcoxon test *P*-values comparing the number of significant motifs, including weak motifs (FIMO *P*-value cutoff of 0.001), found in the regions surrounding in vivo bound and unbound motifs. The bars to the *right* side represent TFs having higher motif counts in their bound sequences, while bars on the *left* side represent TFs having a lower number of motifs in their bound sequences. The height of the bar represents the significance of the differences. The dashed line represents the significance cutoff using the shuffled data. The TFs are colored according to the color code used for TF families: cyan for C2H2 TFs, green for ETS TFs, red for homedomains, and all others in gray. (*D*) Comparison of the PFM similarity scores between sequences surrounding in vivo bound and unbound motifs. The bars on the *right* side represent TFs having higher motif similarity scores in the bound sequences, and bars on the *left* represent TFs having lower similarity scores in the bound sequences. The height of the bar represents the significance of the differences. The dashed line represents the significance cutoff using the shuffled data. The TFs are colored as in panel *C*.

It is well established that TFs belonging to the C2H2 family preferably bind to GC-rich motifs (Choo and Klug 1997; Wolfe et al. 2000), while TFs that belong to the homeodomain family generally bind AT-rich motifs (Gehring et al. 1994; Rohs et al. 2010). Interestingly, for all TFs tested we found a high correlation between the GC content of their binding motif and the preferred GC content in the extended regions surrounding the core motif, both in vitro and in vivo (Fig. 4A). This dependency is exemplified in Figure 4B. As shown, SP1 (a C2H2 protein) and ELF1 (an ETS protein), which bind GC-rich motifs, have a clear preference for high GC environments, while HOXA2 (a homeodomain protein), which binds an AT-rich motif, is surrounded by an AT-rich region. This correlation could be related to the presence of multiple low-affinity binding sites of the same TF (homotypic clusters), which have been shown to be enriched in promoters and enhancers (Lifanov et al. 2003; Sinha et al. 2008; Gertz et al. 2009; Gotea et al. 2010; Ezer et al. 2014; Crocker et al. 2015). We therefore sought to systematically examine the prevalence of homotypic clusters in our data sets. In general agreement with Gotea et al. (2010), we found that bound sequences had, on average, three predicted low-affinity binding sites. When comparing the number of motifs detected in the bound and unbound sequences, we found that for 25 of the 56 TFs, there was a significantly higher number of detected motifs in the extended region around the bound sequences compared with their unbound sequences (Fig. 4C). To examine whether weak motifs are also prevalent in the in vitro data, we used a subset of in vitro TF data sets, for which the TF probes were long enough to harbor at least one binding site not overlapping the core motif. Interestingly, we found that all TFs in the in vitro subset had significantly higher numbers of weak motifs in their bound sequences (Supplemental Fig. 9A), demonstrating intrinsic contributions of low-affinity sites to TF recognition. Interestingly, when we removed all positions with significant similarity to the position frequency matrix (PFM), using different FIMO *P*-value cutoffs for defining significant motifs, the differences in nucleotide content were retained both in vitro and in vivo (Supplemental Figs. 9B, 10D). This raises the possibility that not only do the homotypic clusters represent isolated low-affinity binding sites embedded within genomic content but rather the entire region around the bound motifs is characterized by a unique sequence environment.

**Figure 5.**
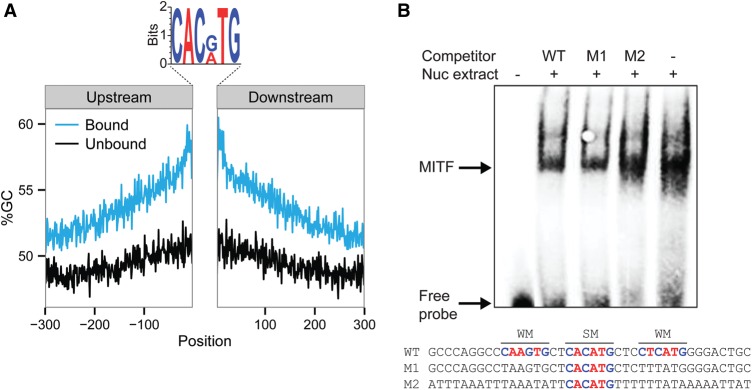
MITF binds to sequences showing overall high similarity to the E-box motif. (*A*) GC content (%GC) upstream of and downstream from motifs in sequences that are bound (blue) and unbound (black) by MITF. Logo of the MITF bound motifs are shown *above*: A and T bases are colored in red; G and C bases in blue. (*B*) EMSA competition assay with probe corresponding to the WT MITF binding region of the human *TRPM1* promoter (WT), probe corresponding to the WT with interruption of the two weak MITF motifs (M1), and probe corresponding to the WT with replacement of all G/C by A/T base pair (M2). Highly expressing MITF melanoma cell (WM3682) nuclear extracts were used as a source of MITF (represented in the Nuc extract row *above*). A biotinylated WT probe was used for the analyses. WT or mutated unlabeled probes as described above were used in the competition analyses. MITF binding probes and free probes are marked with arrows. Probe sequences are shown *below*, where SM indicates strong motif and WM indicates weak motifs.

To better explore the contribution of the overall sequence environment surrounding the motif to TF binding, we experimentally tested the binding of the human TF microphthalmia-associated TF (MITF), employing electrophoretic mobility shift assay (EMSA). MITF belongs to the bHLH family, which naturally binds to the E-box motif, specifically to CACGTG and CACATG (58% GC content on average) (Strub et al. 2011). Since MITF was not represented in our original data, we first analyzed available high-throughput binding data for MITF (Strub et al. 2011) and compared the sequence environment between bound and unbound sequences, both all possessing the MITF motif in open chromatin regions. Consistent with our previous results, our analysis showed that MITF-bound sequences have higher GC frequency around the core motif compared with the unbound sequences possessing the exact same motif (Fig. 5A). To study the contribution of the sequence environment to MITF binding, we tested its binding to a known target sequence (derived from the human *TRPM1* promoter) possessing the E-box core motif surrounded by two weaker motifs (Miller et al. 2004) compared with two designed sequences: In one, we mutated the two weak motifs flanking the core-binding site, while in the other, we changed all G/C to A/T. In all sequences, we retained the core MITF motif. As shown in Figure 5B, mutating the two weak MITF motifs (WM) flanking the core strong motif (SM) showed very similar binding results as for the WT probe, while changing the GC content surrounding the strong motif dramatically reduced MITF binding. This experiment is consistent with our previous results showing that the overall nucleotide content in the environment of the TF motif affects TF binding, irrespective of the presence of weak binding motifs.

Given our previous results, we explored the overall similarity of the environment to the core motif. To this end, we scored each position surrounding the core motif of each TF in the in vivo data by its similarity to the PFM, without using any arbitrary cutoff and compared motif similarity scores between the bound and unbound sequences. We found that the majority of TFs (30 out of 56) had significantly higher similarity scores surrounding the motifs in the bound sequences compared with the unbound sequences (Fig. 4D; for in vitro data, see Supplemental Fig. 9B). Interestingly, when we removed completely all sequences with significant motif matches (not including the core motif), when we removed all positions showing significant similarity to the PFM, or when we compared the number of significant motifs using different cutoffs, the differences between the bound and unbound sequences were retained (Supplemental Figs. 9, 10). Taken together, our results suggest that the previously reported tendency of TF binding sites to be surrounded by low-affinity sites (homotypic clusters) is part of a general tendency of TF binding sites to be embedded within a sequence environment that has overall similar characteristics to the binding motifs (we term the homotypic environment), which contributes to recognition of the cognate binding sites.

### Differences in DNA shape features surrounding bound and unbound TF motifs

It has previously been suggested that DNA flexibility could influence TF binding (Rohs et al. 2010). While specific DNA sequences such as A-tracts have been shown to influence DNA flexibility (Suter et al. 2000), to the best of our knowledge, there is currently no direct way to measure DNA flexibility in a high-throughput manner. Previous studies have suggested that the angle between bases in a base pair (propeller twist) is correlated with DNA flexibility (El Hassan and Calladine 1996; Hancock et al. 2013). We therefore used propeller twist as a proxy of DNA flexibility. To this end, we predicted the propeller twist using our high-throughput method DNAshape (Gordân et al. 2013; Zhou et al. 2013, 2015). Consequently, for each nucleotide position, we compared the predicted values between the bound and unbound sequences, excluding the positions of the core motif. This comparison revealed that the majority of TFs possess significant differences surrounding their motifs both in vitro and in vivo (Fig. 3F; Supplemental Figs. 11, 12). Specifically, we found that TFs belonging to the homeodomain, POU, and forkhead families prefer sequences with an enhanced negative propeller twist, while TFs that belong to the C2H2 and ETS families prefer binding to sequences with a less negative propeller twist. These results were consistent with the knowledge that propeller twist is highly dependent on GC content. Whereas GC-rich sequences tend to have less pronounced propeller twist values, AT-rich sequences tend to have more negative propeller twist values (Hancock et al. 2013). Accordingly, these results support the notion that sequences that contain a bona fide binding site have intrinsic structural features, beyond the core motif, that possibly can be recognized by the TF.

### Binding preferences constrain TF co-occupancy

**Figure 6.**
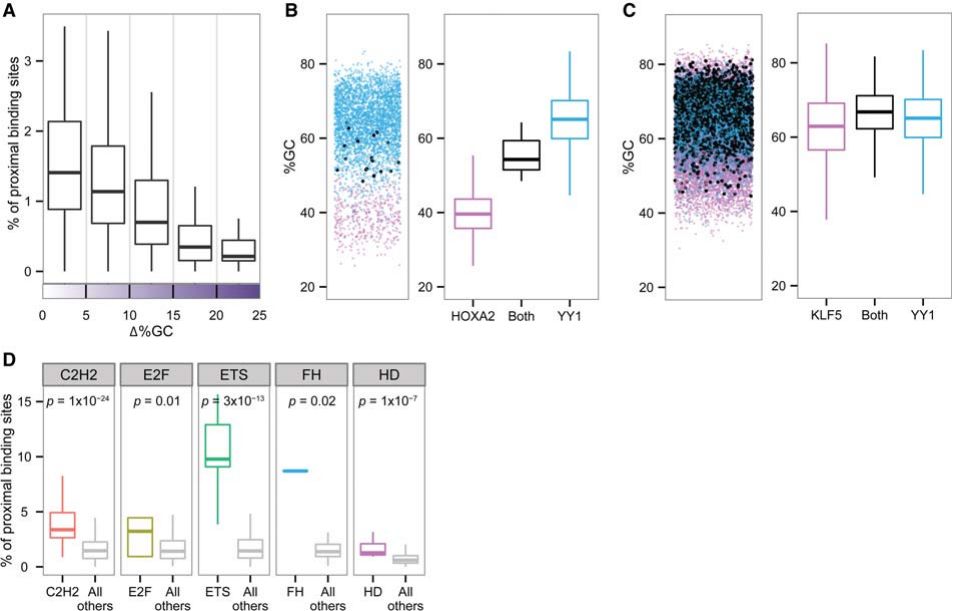
TF co-occupancy frequency. (*A*) Box plots representing the percentage of proximal binding sites relative to the differences in %GC of the motif environment. For each TF pair, the fraction in which one TF binding site occurs in close proximity to a binding site of another TF (*y*-axis) is plotted against the similarity or dissimilarity of the two TFs’ environmental preferences (*x*-axis). Close proximity is defined as 300 bp. (*B*, *left*) Jittered scatterplot showing GC content for each of the sequences bound by HOXA2 (pink), YY1 (blue), and both (black). (*Right*) Box plots representing the distribution of GC content for each of the sequences bound by HOXA2 (pink), YY1 (blue), and both (black). (*C*, *left*) Jittered scatterplot showing GC content for each of the sequences bound by KLF5 (pink), YY1 (blue), and both (black). (*Right*) Box plots representing the distribution of GC content for each of the sequences bound by KLF5 (pink), YY1 (blue), and both (black). (*D*) Box plots representing the percentage of proximal binding sites for each of the five TF families. The data were plotted separately for pairs of TFs from the same family and for pairs of TFs from different families. Wilcoxon test *P*-values indicate significant differences between the groups.

In this study, we found that many TFs have favorable binding environments and that the preference for a specific environment differs between distinct TF families. An intriguing question is how the preference for a specific environment around a TF binding site coincides with the previous observation that TFs tend to bind DNA cooperatively with other TFs (Escalante et al. 2002; Panne et al. 2007; Mann et al. 2009). To answer this question, we measured the co-occupancy frequency in colorectal cancer cells for each TF pair (i.e., the fraction of one TF binding site occurring in proximity to a binding site of another TF) and compared this frequency to the similarity in their GC content preferences around the core motif. We found that pairs of TFs that have very distinct GC content preferences tend to avoid binding close to each other (Fig. 6A). This tendency is exemplified in Figure 6B for the pair YY1 (C2H2 family) and HOXA2 (homeodomain family). As shown, these TFs have very different GC content preferences (YY1 prefers binding to a GC-rich environment; HOXA2 prefers regions with low GC content). Here, we show that YY1 and HOXA2 target sites are rarely found in close proximity, with only 0.3% of YY1 binding sites found close to those of HOXA2 and 2% of all HOXA2 binding sites found close to those of YY1. Consistently, we found that pairs of TFs that have similar GC preferences are found more frequently to co-occupy the DNA. Examples are YY1 and KLF5, which are two C2H2 TFs that prefer binding to regions with high GC content (Fig. 6C). We found that 41% of all YY1 binding sites are located close to KLF5 binding sites. In agreement with this, we found that pairs of TFs from the same family have more proximal binding sites compared with pairs of TFs belonging to two different families (Fig. 6D). Our results suggest that a dependency exists between environmental preferences and the tendency of TFs to bind in proximity to each other.

### The motif environment contributes to prediction of TF binding sites

**Figure 7.**
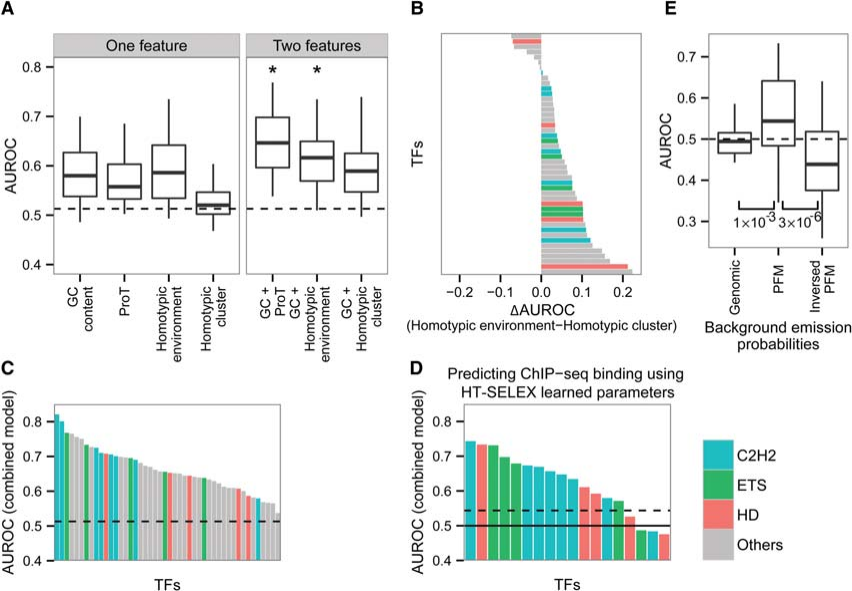
Predicting bound and unbound TF motifs. (*A*) L2-regularized multiple linear regression models based on one or two features in vivo. The features characterizing the average GC content, the average propeller twist (ProT), the average PFM similarity scores (homotypic environment), and the sum of all significant PFM similarity scores (using FIMO *P*-value cutoff of 0.001; homotypic cluster). All features were extracted from 300 bp upstream of and downstream from the core motif, excluding the core motif. Box plots represent the distribution of the AUROC for all TFs using one or two features. The dashed line represents the maximum AUROC obtained using randomly shuffled data. Asterisks are shown for features in which the AUROC obtained using the two-feature model is significantly higher than the AUROC obtained using each feature separately. (*B*) For each TF, comparison of the AUROC obtained using the homotypic environment model and the homotypic cluster model. The TFs are colored according to the color code used for TF families: cyan for C2H2 TFs, green for ETS TFs, red for homedomains, and all others in gray. (*C*) AUROC values for each of the TFs, employing a model that incorporates the best preforming features: GC content, propeller twist, and homotypic environment. Dashed line represents the maximum AUROC obtained using randomly shuffled data. (*D*) AUROC of the combined model that was trained using the in vitro data and was tested on the in vivo data. Dashed line represents the maximum AUROC obtained using randomly shuffled data. Solid line shows AUROC of 0.5. (*E*) AUROC of the HMMs using different emission probabilities for the background state: the genomic nucleotide frequency, average nucleotide frequency of the PFM, and the inversed average nucleotide frequency of the PFM. Wilcoxon test *P*-values are shown *below*. The dashed line represents AUROC of 0.5.

Given our results showing that motif environments differ significantly between bound and unbound motifs, we sought to assess whether environmental properties could help to discriminate between bound and unbound sequences. To this end, we employed L2-regularized multiple linear regression (MLR) models that incorporated different environmental features surrounding each motif as described in the Methods. Since it has been shown that the motif strength (match to PFM) is correlated with TF binding (Gertz et al. 2013; Madsen et al. 2014; Sherwood et al. 2014), we used bound and unbound sequences harboring the same motif strength distribution, thus completely removing the effect of the motif strength. As a first step, we trained four different models using features extracted from sequences surrounding the core motif, excluding the core motif and two positions from each side: Model 1, using the average GC frequency (GC content); Model 2, using the average propeller twist (propeller twist); Model 3, using the average motif similarity scores (homotypic environment); and Model 4, using the summary of all motif scores above a FIMO *P*-value cutoff of 0.001 (homotypic clusters; for details, see Methods). We evaluated the model performance using the area under the receiver operating characteristic (AUROC) and found that models that use only a single environmental feature have a moderate discriminative power, with an average AUROC of 0.58, 0.57, 0.58, and 0.53 for GC content, propeller twist, homotypic environment, and homotypic clusters, respectively (Fig. 7A). By comparing the performance of the homotypic environment model to the homotypic cluster model, we found that for most TFs the homotypic environment feature performed better (average AUROC of 0.59 compared with 0.53) (Fig. 7B), emphasizing again that bound motifs have a unique sequence environment that resembles the motif itself. To further assess whether the environmental features are also relevant in vitro, we repeated the above analyses using bound and unbound sequences from the HT-SELEX experiments, which have similar distributions of motif match scores (as described for the in vivo data). Here again, we found that for bound and unbound sequences with similar motifs, a single environmental feature can aid in binding prediction (average AUROC of 0.54, 0.53, 0.53, and 0.51 for GC content, propeller twist, homotypic environment, and homotypic clusters, respectively) (Supplemental Fig. 13A). However, in respect to the prediction accuracy for the in vivo data, the contribution of the environment in the in vitro data was smaller (Supplemental Fig. 13B), possibly due to shorter lengths of the HT-SELEX sequences (22 nt on average) compared with 300 bp upstream of and downstream from the motif using the genomic environments.

In order to assess the interdependency between the GC content and the three other features, we trained three additional models combining GC content with propeller twist, homotypic environment, or homotypic clusters and found that adding a second feature to the GC content significantly improved the predictions (average AUROC improvement of ∼8%) (Fig. 7A), supporting our findings that propeller twist, homotypic environment, and homotypic clusters have an additional and independent effect on binding from that of the GC content. We also found for the in vitro data, in general agreement with the in vivo data, that adding GC content as a second feature to a model that uses either propeller twist, homotypic environment, or homotypic clusters significantly improves binding prediction (AUROC improvement of ∼3%, 3%, and 7%, respectively) (Supplemental Fig. 13A). We next combined the three best-performing features (GC frequency, propeller twist, and homotypic environment) into one model, which further improves binding predictions (average AUROC of 0.66) (Fig. 7C), showing that the addition of propeller twist to GC frequency and homotypic environment improves binding prediction for most TFs (Supplemental Fig. 14). Notably, for some TFs we found that this model highly discriminates between bound and unbound motifs (Fig. 7C). For example, for SP1 and YY1 (members of the C2H2 family), a model combining all three features that characterize motif environments, without any information from the core motif, resulted in AUROC values of 0.8 or more.

Next we asked whether TF preferences learned using in vitro data could also be applied to binding predictions in vivo. To this end, we used the combined model trained using the HT-SELEX data in order to predict in vivo bound and unbound sequences, focusing only on TFs belonging to the homeodomain, ETS, and C2H2 families. We found that a model trained using in vitro data contributed to the in vivo prediction for 14 out of 18 TFs (average AUROC 0.62) (Fig. 7D).

In addition to the MLR, we applied a hidden Markov model (HMM) approach to predict bound sequences. In brief, the HMM approach scores each sequence by the log likelihood ratio, which reflects the probability of a sequence to be generated by sampling from the PFM or different backgrounds (as described below) versus the probability of it being generated solely by a genomic background. This HMM model requires no predefined motif cutoff and therefore accounts for high- and low-affinity sites, does not require data for training, and, thus, avoids overfitting. We have found that an HMM model that uses the average nucleotide frequencies from the TF's PFM as the background probability performed significantly better than the HMM that uses the genomic nucleotide frequencies as the background probability (Wilcoxon *P*-value = 0.001, improvement for 71% of TFs), which emphasizes the importance of the homotypic environment in modeling TF binding sites (Fig. 7E; Supplemental Figs. 15, 16). For a comparison, we created a third model, this time swapping the nucleotide background probabilities, and found a significantly lower performance for the prediction of bound sequences (Wilcoxon *P*-value = 3 × 10^−6^). Overall, the results from the MLR and the HMM models indicate that the motif environments hold crucial information and, presumably, contribute to the recognition of the binding site by the TF.

## Discussion

Over the past decade, an extensive amount of information on binding preferences of TFs has been accumulated from in vitro and in vivo high-throughput binding assays (Harbison et al. 2004; Berger et al. 2008; Noyes et al. 2008; Badis et al. 2009; Wei et al. 2010; The ENCODE Project Consortium 2012; Enuameh et al. 2013; Jolma et al. 2013; Nakagawa et al. 2013; Yan et al. 2013). Whereas these studies allow deriving consensus binding sites as well as PFMs of hundreds of TFs, both are insufficient for accurate identifications of the targets of a given TF within the genome. In this study, we sought to search for intrinsic features that discriminate bound from unbound sequences that possess a cognate binding motif. In an attempt to identify the features of sequences that are bound preferably by a given TF, we analyzed recently published HT-SELEX data for 239 TFs (Jolma et al. 2013) in which epigenetic effects are controlled. Furthermore, we extracted in vivo binding data from 56 ChIP-seq experiments (Yan et al. 2013), examining regions of open chromatin to avoid biases between bound and unbound sequences due to chromatin accessibility of the target sequence. By use of both approaches, we demonstrated that the information encoded in the regions surrounding the binding motifs allows distinguishing bound from unbound motif-containing sequences. These results were observed consistently for the vast majority of TFs studied, showing that this is a widespread phenomenon. Furthermore, we found that these preferences are family specific; homeodomain TFs prefer binding to AT-rich regions, whereas C2H2 and ETS prefer regions of high GC content, both in vivo and in vitro. Interestingly, C2H2 zinc finger and homeodomain TFs, which are the two largest TF families in eukaryotes (Vaquerizas et al. 2009), have opposite nucleotide preferences at the regions surrounding the core motif.

A recent ENCODE study (The ENCODE Project Consortium 2012) suggested that most TFs bind to GC-rich regions. This is consistent with the fact that in vivo, most TFs bind to accessible DNA regions (John et al. 2008; Song et al. 2011; Thurman et al. 2012), which tend to have higher GC content compared with the rest of the genome (Fenouil et al. 2012). By restricting the analysis to accessible DNA regions, we were able to control GC differences due to DNA accessibility, further discovering that most TFs have specific DNA preferences beyond their preference for accessible regions. Transcriptional regulation is believed to be a highly dynamic and complex process carried out at multiple levels (Levo and Segal 2014; Slattery et al. 2014; Voss and Hager 2014). The basic level required for binding involves chromatin accessibility, which can roughly divide the genome into closed chromatin (heterochromatin), which is inaccessible to the majority of TFs, and accessible regions (euchromatin), which are transcriptionally active regions where most TF binding occurs (Grewal and Moazed 2003; Huisinga et al. 2006). However, there are examples of so-called pioneer TFs that prefer binding to closed regions (Barozzi et al. 2014). Here, we suggest an additional level that is encoded by the local environment, which may help to direct the TFs to their binding regions. Finally, the undeniably dominant level involves the recognition of specific short DNA motifs by a given TF.

We found that TF sequence preferences reach far beyond the core motifs and their direct flanks, which were previously shown to contribute to TF binding preferences (Gordân et al. 2013; Afek et al. 2014; Levo et al. 2015). An intriguing question is how the protein can identify the unique environment so far beyond its binding site. Based on our analysis, which was conducted for TFs from several TF families, we found an overall strong dependency between nucleotide composition of the motif and its environment. These dependencies, which we found for the vast majority of TFs, are consistent with early genomic observations showing that TATA-box–containing promoters are generally AT rich, while TATA-less promoters have a high GC content (Sandelin et al. 2007; Yang et al. 2007). It was previously suggested that the homotypic clusters are important components of the regulatory elements and might have a functional advantage in facilitating the recruitment of TFs (Lifanov et al. 2003; Sinha et al. 2008; Gotea et al. 2010; Ezer et al. 2014; Crocker et al. 2015). We conducted a systematic, TF binding site–based examination, measuring the prevalence of homotypic clusters for 21 and 56 TFs from different families, employing both in vitro and in vivo data sets, respectively. We found, in agreement with previous studies conducted on individual TFs, that the bound sequences of the majority of TFs are significantly enriched in homotypic clusters compared with sequences found in unbound regions, (Zhang et al. 2006; Gotea et al. 2010). However, while the regions surrounding TF binding site peaks show some evolutionary conservation (Håndstad et al. 2011, 2012), previous studies show that even when deleting some of the weak motifs surrounding the binding sites, often there are no detectable changes to gene expression (Driever and Nüsslein-Volhard 1989; Doniger et al. 2005; Estella et al. 2008). Here we found that weak binding motifs are usually found embedded within an overall sequence environment that resembles the core motif of the TF. We propose that the tendency of TF binding motifs to be found within an overall homotypic environment may have been selected in evolution to narrow down the search space of a given TF and increase the thermodynamic probability of binding to a site. We showed that in addition to the preference of TFs to bind to regions with similar nucleotide content compared with their binding motif, the sequence-dependent DNA shape of the motif environment might also play a role in TF recognition (Rohs et al. 2009, 2010; Zhou et al. 2015). By use of our high-throughput DNA shape prediction method (Zhou et al. 2013), we analyzed the DNA propeller twist and noticed that differences in propeller twist, which are consistent with differences in GC content, can be found as far as 300 bp from the core-binding motif. Possibly, the DNA features surrounding the binding site may contribute to the attraction of TFs that belong to different families to their cognate binding sites. Furthermore, other possible mechanisms could explain the differences between the sequence environments found in the in vivo data, such as cooperative binding and dynamic time-dependent changes in the chromatin state.

It is well established that the regulation of transcription is achieved by complex interactions of different TFs that bind close to each other on the DNA. Our analyses show that a dependency exists between the environmental preferences of the TFs and their tendency to bind close to each other, which suggests that the environmental preferences of each TF restrict the binding of other neighboring factors. The implication of such a constraint is that TFs from the same family could co-occupy more easily compared with TFs belonging to different families. A well-known example of TF family–specific cooperativity is HOX proteins, which are homeodomains that bind DNA with cofactors from the same family in order to evoke their binding specificity (Slattery et al. 2011; Abe et al. 2015).

Finally, by employing two different prediction algorithms, we show that the DNA environment alone (excluding information from the motif itself) can help distinguishing between bound and unbound TF motifs. Currently, motif-scanning tools that consider local background models to compensate for regional biases in nucleotide composition are available. However, in these approaches, a GC-rich motif residing in a GC-rich region would receive a lower score (compared with a GC-rich motif in an AT-rich region) to remove false-positive weak motifs around the true binding site. Based on our results, we suggest an adjustment to the local background models, in which the most significant motif within a region is promoted when residing in an environment with a GC content that is similar to the motif. That could improve prediction performance by reducing the false-negative predictions introduced by the local background models.

In summary, our analyses further support the emerging view that regions surrounding TF binding motifs, which tend to be overlooked in characterizations of TF binding due to their low sequence information, might have an important contribution to TF binding both in vitro and in vivo.

## Methods

### Data collection

#### In vitro data collection and motif alignment

HT-SELEX data were collected from a study of 241 unique TFs (Jolma et al. 2013). TF data sets with less than 1000 sequences after alignment (see below) were removed, resulting in data sets for 239 TFs (Supplemental Table 1). In cases for which there was more than one HT-SELEX experiment, the experiment with the higher sequence count was selected. The TFs were grouped into 19 Pfam structural families (Finn et al. 2014). TF families with fewer than 10 members were grouped under “Others.” For the bound sequences, we used the final selection round as previously described (Jolma et al. 2013). For the unbound sequences, we collected two data sets: one using sequences from “round zero” (the initial pool of random oligonucleotides) and a second using sequences from “round minus one” (one round before the selected round). To prevent biases resulting from differences in sample size, we randomly selected a subset of the bound or unbound pools to match the size of each other. Orenstein and Shamir (2014) recently described the biases that might occur in HT-SELEX experiments. To test and control for possible biases, several analyses were carried out as described in detail in the Supplemental Information. PFMs were collected for each TF (Jolma et al. 2013) and used to search and align the bound and unbound sequences using FIMO (Grant et al. 2011). In cases of palindromic motifs, we used both strands. In cases where more than one motif was found per sequence, we chose the position with the highest score. Further, if more than one motif with the same (highest) score was detected within a sequence, the sequence was discarded. The length of the probes varied from 14 bp (three TFs), 20 bp (194 TFs), 30 bp (23 TFs), to 40 bp (19 TFs). Since different TFs have variable probe lengths, the GC frequency analysis was conducted consistently for 10 bp upstream of and downstream from the TF core-binding motif.

#### In vivo data collection and motif alignment

ChIP-seq data for 71 human TFs from colorectal cancer cells along with their published IUPAC sequence motifs were extracted from a recent study (Yan et al. 2013). All appearances of the IUPAC motif seed, allowing for one mismatch, were collected from the TF ChIP-seq peaks (an approach based on the method described by Berger et al. 2006). The sequences collected were further used to construct a PFM that enabled a refined motif search and alignment method. The final PFM was used to search and align the bound and unbound sequences using FIMO (Grant et al. 2011). In cases of palindromic motifs, both strands were used. In cases where the motif was found more than once in a sequence, the position with the highest FIMO motif score was used. If more than one motif with the same (highest) score was detected within a sequence, that sequence was discarded. In cases where the peak of the motif distribution did not coincide with the ChIP-seq peak summit, the data for that TF were discarded. The final set included 56 TFs that were assigned to 21 Pfam (Finn et al. 2014) families. TF families with fewer than four members were grouped under “Others.” For the bound sequences, we used sequence motifs found in ChIP-seq peaks. For the unbound motifs, we used sequences containing the motifs that were located outside the ChIP-seq peaks. To prevent biases resulting from differences in sample size, we randomly selected a subset of the bound or unbound pools to match the size of each other. Promoter regions were defined using RefSeq genes (Pruitt et al. 2014), using 1000 bp upstream of and downstream from the TSS. Human MITF ChIP-seq data from melanoma cells were extracted from a separate study (Strub et al. 2011). The E-box motif, specifically CACGTG and CACATG, which was characterized as MITF preferred motif (Strub et al. 2011), was used to align MITF ChIP-seq peaks.

### Characterization of open and closed chromatin

DNase I hypersensitivity data from colorectal cancer cells were extracted from the experiment described previously (Yan et al. 2013) and were analyzed using the Hotspot tool (John et al. 2011). DNase I hypersensitivity data from melanoma cells were extracted according to a method described previously (Marzese et al. 2014) (GEO accession number GSM1008599). DNase I hypersensitive sites were defined as open chromatin regions.

### DNA shape analysis

Propeller twist analysis was conducted using DNAshape, our high-throughput DNA shape prediction method (Zhou et al. 2013), which infers structural features from a library of all-atom Monte Carlo simulations using a sliding pentamer window. The predicted average values of propeller twist were obtained for each nucleotide position of the aligned sequences.

### Comparative analysis of the bound and unbound sequences

A comparison of the features between the bound and unbound sequences was conducted for each position in the aligned sequences using the one-sided Wilcoxon signed-rank test. To correct for multiple testing, we used false-discovery rate (FDR) *q*-values (Storey and Tibshirani 2003). The Δ[−log(*q*-value)] comparing the hypothesis that bound > unbound versus the alternative unbound > bound was assigned to each position. A negative Δ[−log(*q*-value)] was assigned to positions at which the unbound sequences had significantly higher values in the feature examined than the bound. A positive Δ[−log(*q*-value)] was assigned to positions at which the bound sequences had significantly higher values in the examined feature than the unbound sequences. A TF was defined as having a preference for a specific feature if it had at least five positions with significant differences (*q*-value ≤ 0.05).

### Comparative analysis of homotypic clusters and homotypic environment

A comparison of homotypic clusters between the bound and unbound sequences was done by counting the number of detected motifs using three FIMO *P*-value cutoffs: ≤0.001, 0.001–0.05, and 0.05–0.1. A homotypic environment comparison was conducted using a sliding window approach applied to each of the bound and unbound sequences in which the size of the window is the length of the motif. The sequence in each window was assigned a similarity score using the log-odds scores of the PFM. The positions of the core motif were removed from both the calculations in order to prevent motif biases. A comparison between scores of the bound and unbound sequences was made using the Wilcoxon signed-rank test as described above. For the in vitro analysis, we used a subset of TFs in which their probe length was long enough to harbor at least one binding site that is at a distance of at least 2 nt from the core motif.

### Calculating co-occupancy of TF binding sites

The frequency of co-occupancy of TF binding sites was calculated for all possible pairs of TFs in the in vivo data set in colorectal cancer cells. This was conducted by collecting the motif-containing peaks for each given TF and calculating the frequency of the appearances of binding sites of all other TFs within a distance of 300 bp from the motif-containing peak. Since TFs belonging to similar families often have similar binding motifs, overlapping motifs were discarded. Consequently, each of the co-occupancy frequencies was compared with the similarity in GC preferences of the pair. The latter was conducted by calculating the average *q*-value of the GC preferences over all positions for each TF and calculating the ratio of the two averages between each of the TF pairs.

### Predicting binding motifs

#### MLR scoring scheme

Four L2-regularized MLR models were trained using one of the four different features: (1) To study the contribution of GC content, the average GC frequency over 300 bp upstream of and downstream from the core motif was considered; (2) to study the contribution of propeller twist, the average propeller twist over 300 bp upstream of and downstream from the core motif was employed; (3) to study the contribution of homotypic environment, the average PFM similarity scores over each window 300 bp upstream of and downstream from the core motif was considered; and (4) for the contribution of homotypic clusters, the sum of all PFM scores above a FIMO *P*-value score of 0.001, over each window along the 300 bp upstream of and downstream from the core motif, was taken. To measure the predictive power of each of the four models, a 10-fold cross-validation was performed. λ (the penalty parameter) was learned from the data using an embedded 10-fold cross-validation on the training set. The AUROC was used to assess the accuracy of the model in predicting the bound and unbound sequences. The AUROC values generated from the models were compared with three additional models, each with two features: (1) GC content and propeller twist, (2) GC content and homotypic environment, and (3) GC content and homotypic clusters. The three most predictive features—GC content, propeller twist, and homotypic environment—were further employed in a third model (combined model). For comparison, sequences were shuffled between the bound and unbound groups, and the combined parameters were retrained. The maximum AUROC value of the model based on shuffled sequences was used as an empirical significance cutoff. A similar approach was used to predict in vitro bound and unbound sequences. To evaluate the ability of predicting in vivo bound sequences using in vitro data, parameters were learned for three TF families: homeodomain, ETS, and C2H2 using in vitro data. Tenfold cross-validation was performed for each of the three families in order to learn λ. The learned coefficients of the three features (GC content, propeller twist, and homotypic environment) were then used to predict bound sequences for each TF from one of the three families in the in vivo data.

#### HMM scoring scheme

A statistical probability model characterizing the space of all valid binding configurations was used. The model is composed of a state modeling the TF binding site (motif state) and a state modeling the background surrounding the TF binding site (Supplemental Fig. 17). The emission probability of the motif state corresponded to the nucleotide frequency extracted from the PFM. The background state is characterized by a PFM of a length of one (the emission probabilities for the background state are described below). The transition probability of moving from the motif state to the background state was arbitrarily set to 0.99 as according to the method previously described (Hoffman and Birney 2010). To allow occurrences of the motif in both directions (for binding sites on both strands), the strand bias of each motif was precalculated by extracting the frequency of the motif (using a FIMO threshold of *P*-value = 0.001) in the bound sequences in both directions; the strand bias was used for defining the motif transition probability. The log likelihood ratio was calculated for each sequence, reflecting the likelihood of a given sequence to be generated from the HMM model that uses both the motif and the background states (described below), as opposed to being generated solely by a fixed genomic background state, where the emission probabilities of each nucleotide are taken from the unbound sequences of each TF separately. Three different HMM models were implemented using different emission probabilities for the background state: (1) the genomic background described above, (2) the average PFM AT/GC frequencies, and (3) the inversed average PFM AT/GC frequencies.

### Electrophoretic mobility shift assay

Nuclear extracts were prepared using a NE-PER nuclear and cytoplasmic extraction kit (Pierce) according to the manufacturer's instructions. The MITF biotin-labeled DNA probes spanning MITF binding sites were obtained from IDT. Binding reactions of 10 μg of nuclear lysates and 0.02 pmol of labeled double-stranded DNA probe were performed for 20 min on ice using a LightShift chemiluminescent EMSA kit (Pierce) according to the manufacturer's instructions. Competition analyses were performed with an excess (30 pmol) of unlabeled probes. Samples were resolved by 5% PAGE in 0.5× TBE buffer (45 mM Tris borate, 1 mM EDTA) transferred to nitrocellulose membranes. Labeled DNA was visualized with the ECL system (Pierce). The super-shift assay is shown in Supplemental Figure 18. Probe sequences for the WT and the mutated MITF binding sites are listed in Figure 5B. The WT probe was derived from the *TRPM1* promoter, which was shown to bind MITF (Miller et al. 2004). In M1, mutations were introduced to disrupt MITF low-affinity binding sites. In M2, all G and C bases were replaced by A and T bases, respectively.

## Supplementary Material

Supplemental Material
