# Supplementary material for: A widespread role of the motif environment in transcription factor binding across diverse protein families
Source: Genome Res. 2015 Sep;25(9):1268–80. doi: 10.1101/gr.184671.114 (PMC4561487; doi:10.1101/gr.184671.114)
Supplement: Supplemental Material [file supp_25_9_1268__index.html]

A widespread role of the motif environment in transcription factor binding across diverse protein families — Supplemental Material 

# A widespread role of the motif environment in transcription factor binding across diverse protein families

## Supplemental Material

**Files in this Data Supplement:**

- Supplemental Material.pdf
- Supp Tables.xlsx
