# Supplementary material for: A widespread role of the motif environment in transcription factor binding across diverse protein families
Source: Genome Res. 2015 Sep;25(9):1268–80. doi: 10.1101/gr.184671.114 (PMC4561487; doi:10.1101/gr.184671.114)
Supplement: Supplemental Material [file supp_gr.184671.114_Supplemental_Material.pdf]

SUPPLEMENTAL MATERIAL

A widespread role of the motif environment in transcription factor binding across diverse protein families

Iris Dror[1,2], Tamar Golan[3], Carmit Levy[3], Remo Rohs[2,4] and Yael Mandel-Gutfreund[1,4]

[1] Faculty of Biology, Technion – Israel Institute of Technology, Technion City, Haifa 32000, Israel
[2] Molecular and Computational Biology Program, Departments of Biological Sciences, Chemistry, Physics, and Computer Science, University of Southern California, Los Angeles, CA 90089, USA
[3] Department of Human Genetics and Biochemistry, Sackler Faculty of Medicine, Tel Aviv University, Tel Aviv 69978, Israel

[4] Corresponding authors: rohs@usc.edu (RR) and yaelmg@tx.technion.ac.il (YMG)

In vitro HT-SELEX data analysis

For the *in vitro* analysis we used HT-SELEX data from (Jolma et al. 2013). A recent paper (Orenstein and Shamir 2014) discussed the possible biases in HT-SELEX datasets. In order to account for any possible biases that could influence our results, we conducted the following steps:

1. We removed all oligonucleotides containing five consecutive A, C, T, or G nucleotides, as those have been reported (Orenstein and Shamir 2014) to be overabundant in the HT-SELEX data, especially in round 0-2.
2. We filtered out oligonucleotides, which did not include the known published motif as such oligonucleotides (defined as ”false oligos”) were found to be common in the HT-SELEX experiment (Orenstein and Shamir 2014).
3. It has been reported (Orenstein and Shamir 2014) that each of the rounds exhibits a bias for A over C over G over T, and for A+T over G+C. This bias was observed in all of the SELEX rounds, emphasizing the importance of the comparison between the rounds. In our analysis we compared the selected round to the “minus one” round, and to the “initial round”. Since in our study we conducted only comparative analyses, such biases are not expected to influence our results. Furthermore, it has been reported that the percentages of A and C remained constant in all rounds. Notably, when comparing A or C percentages between the selected round and the “minus one” round, concentrating only on the plus strand, the differences remained significant.

Sample size and multiple testing

To ensure that the significant differences found in GC content between the bound and the unbound sequences (Fig. 2A and Fig. 3A) are not due to sample size, multiple testing, or motif combinations, we generated four control tests.

1. As mentioned in the main text, we used position frequency matrices (PFM) to define sequences containing the TF motif. While this procedure resulted in both groups having the TF binding motif, the bound and unbound pools had differences in motif composition. To deal with these differences, we randomly selected a subset of the bound and the unbound sequences so that both groups have the same motif composition and compared GC content between the bound and the unbound groups as described in the Methods. This analysis yielded similar results compared to the original results, supporting that the significant differences observed in our analysis were not a result of the differences in the motifs between the bound and unbound sets (Supplemental Fig. 1 and 6).

2. For the *in vitro* data, we considered sequences from the initial pool of random oligonucleotides as unbound sequences and repeated the same analysis. Whereas this pool is expected to be more enriched with unbound sequences (compared to the "minus one" round), this group contained substantially less sequences, making it harder to observe statistically significant differences. Nonetheless, the analysis yielded similar results compared to the results obtained using the "minus one" round (Supplemental Fig. 2). The differences in binding strength between the different rounds were described in detail in (Orenstein and Shamir 2014).

3. We randomly shuffled the sequences between the bound and the unbound pools (keeping the size of each pool constant), and found no significant differences (q-value < 0.05) in GC content (Supplemental Fig. 5).

Supplemental Fig. 1

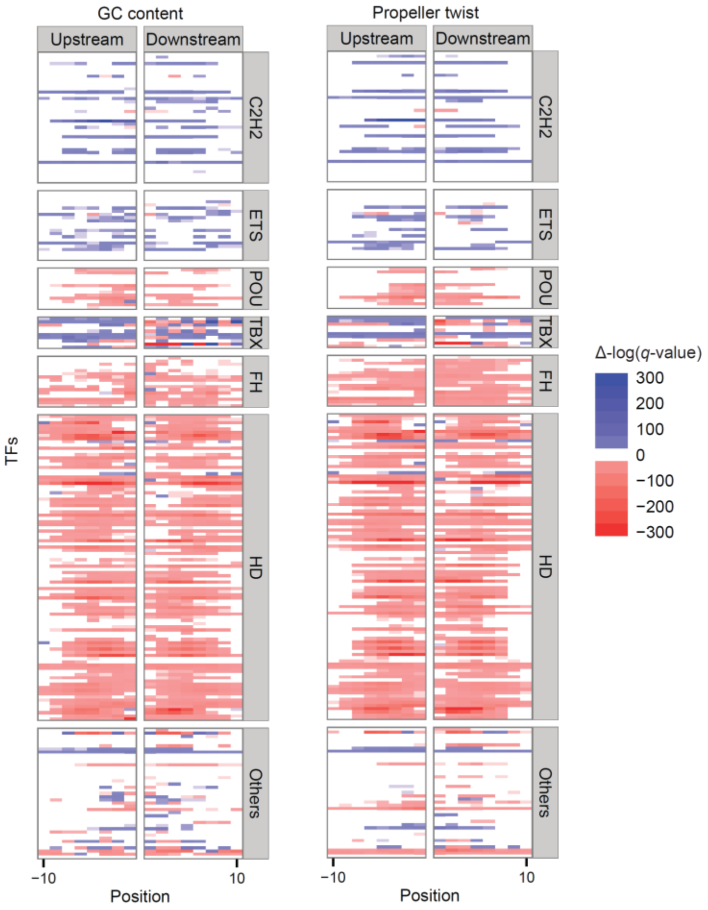

Supplemental Fig. 1: Differences in features of the regions surrounding TF motifs in bound and unbound sequences that have similar motif composition extracted from *in vitro* data. Heat map representing the differences in GC content (*left*), and propeller twist (*right*) 10 bp up- and downstream of the core motifs, whereby red indicates positions at which the respective feature value was lower in the bound motifs, and blue represents positions at which the respective feature value was higher in the bound compared to the unbound motifs (the color intensity represents the significance). The TFs were grouped by the different TF families (FH for forkhead, HD for homeodomain). The positions correspond to the core-binding motif.

Supplemental Fig. 2

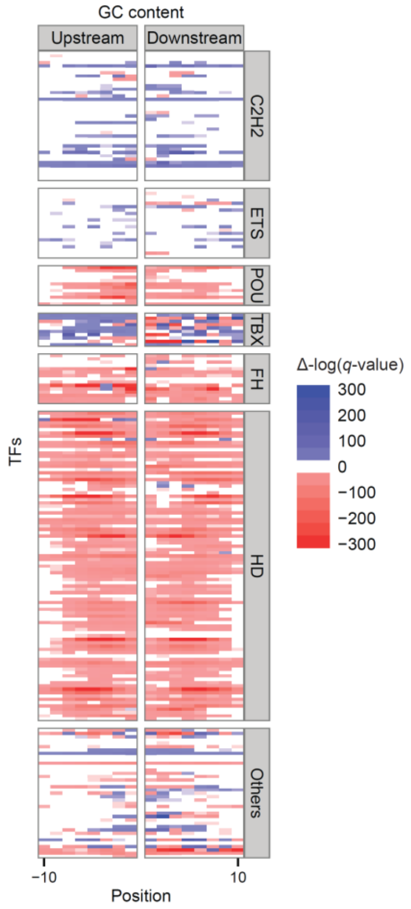

Supplemental Fig. 2: Differences in features of the regions surrounding TF motifs, between motifs found in bound and the initial pool of random oligonucleotides extracted from *in vitro* data. (A) Heat map representing the differences in GC content 10 bp up- and downstream of the core motifs, whereby red indicates positions at which the respective feature value was lower in the bound motifs, and blue represents positions at which the respective feature value was higher in the bound compared to the unbound motifs (the color intensity represents the significance). The TFs were grouped by the different TF families (FH for forkhead, HD for homeodomain). The positions correspond to the core-binding motif.

Supplemental Fig. 3

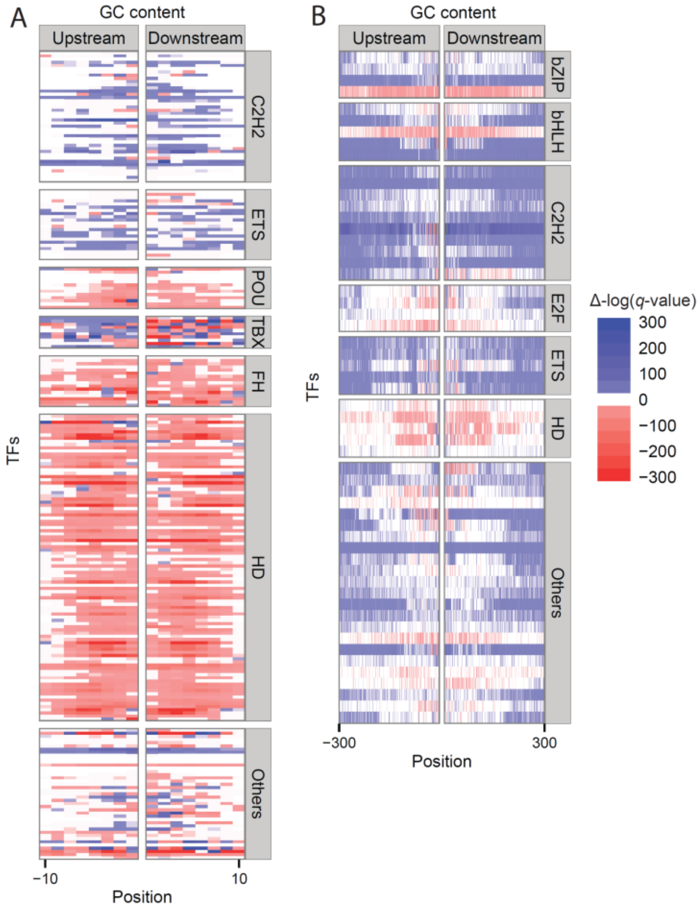

Supplemental Fig. 3: Differences in GC content of the regions surrounding TF motifs in bound and unbound sequences extracted from *in vitro* (A) and *in vivo* (B) data. Heat maps representing the differences in GC content up- and downstream of the core motifs. *p*-values calculated using Chi-squared test, whereby red indicates positions at which the GC content was lower in the bound motifs, and blue represents positions at which the GC content was higher in the bound compared to the unbound motifs (the color intensity represents the significance). The TFs were grouped and colored by the different TF families (FH for forkhead, HD for homeodomain). The positions correspond to the core-binding motif.

Supplemental Fig. 4

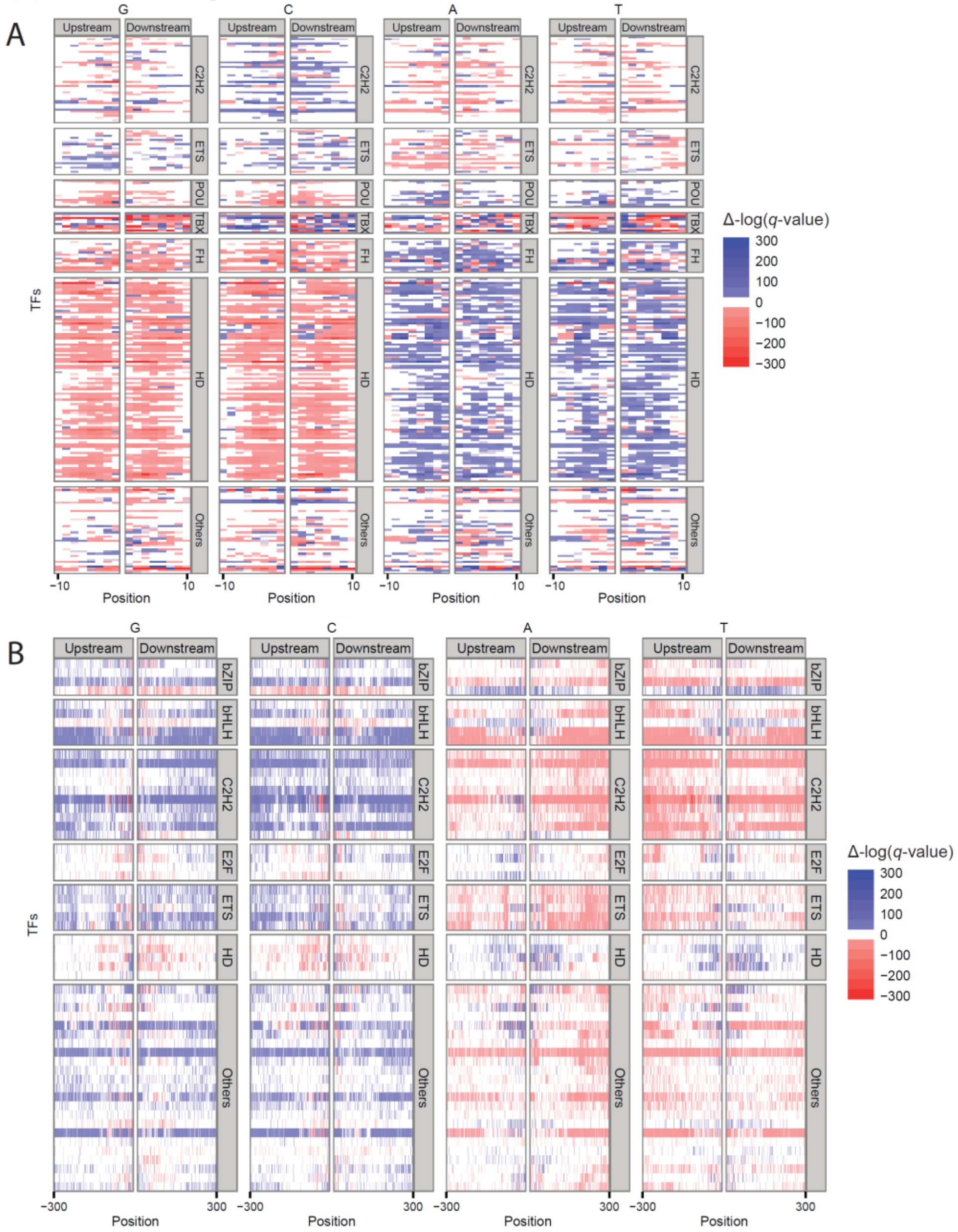

Supplemental Fig. 4: Differences in nucleotide content between the regions surrounding TF motifs in bound and unbound sequences extracted from *in vitro* (A) and *in vivo* (B) data. Heat map representing the differences in G, C, A, and T up- and downstream of the core motifs, whereby red indicates positions at which the respective feature value was lower in the bound motifs, and blue represents positions at which the respective feature value was higher in the bound compared to the unbound motifs (the color intensity represents the statistical significance). The TFs were grouped by the different TF families (FH for forkhead, HD for homeodomain). The positions correspond to the core-binding motif.

Supplemental Fig. 5

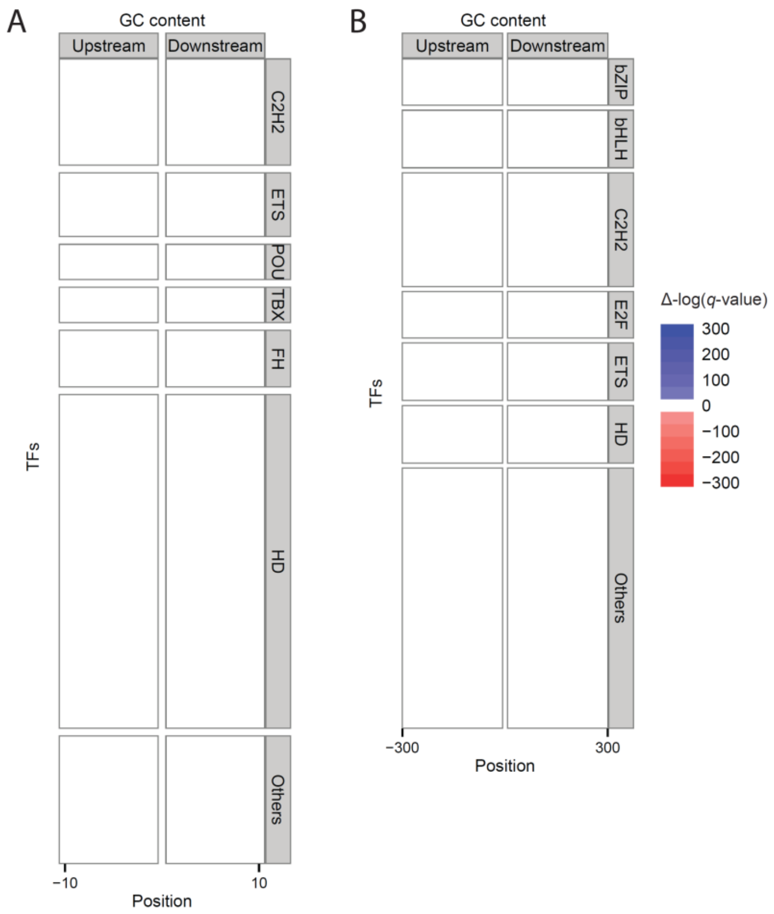

Supplemental Fig. 5: Differences in GC content of the regions surrounding TF motifs in randomly shuffled bound and unbound sequences extracted from *in vitro* (A) and *in vivo* (B) data. Heat map representing the differences in GC content up- and downstream of the core motifs. The TFs were grouped by the different TF families (FH for forkhead, HD for homeodomain). The positions correspond to the core-binding motif.

Supplemental Fig. 6

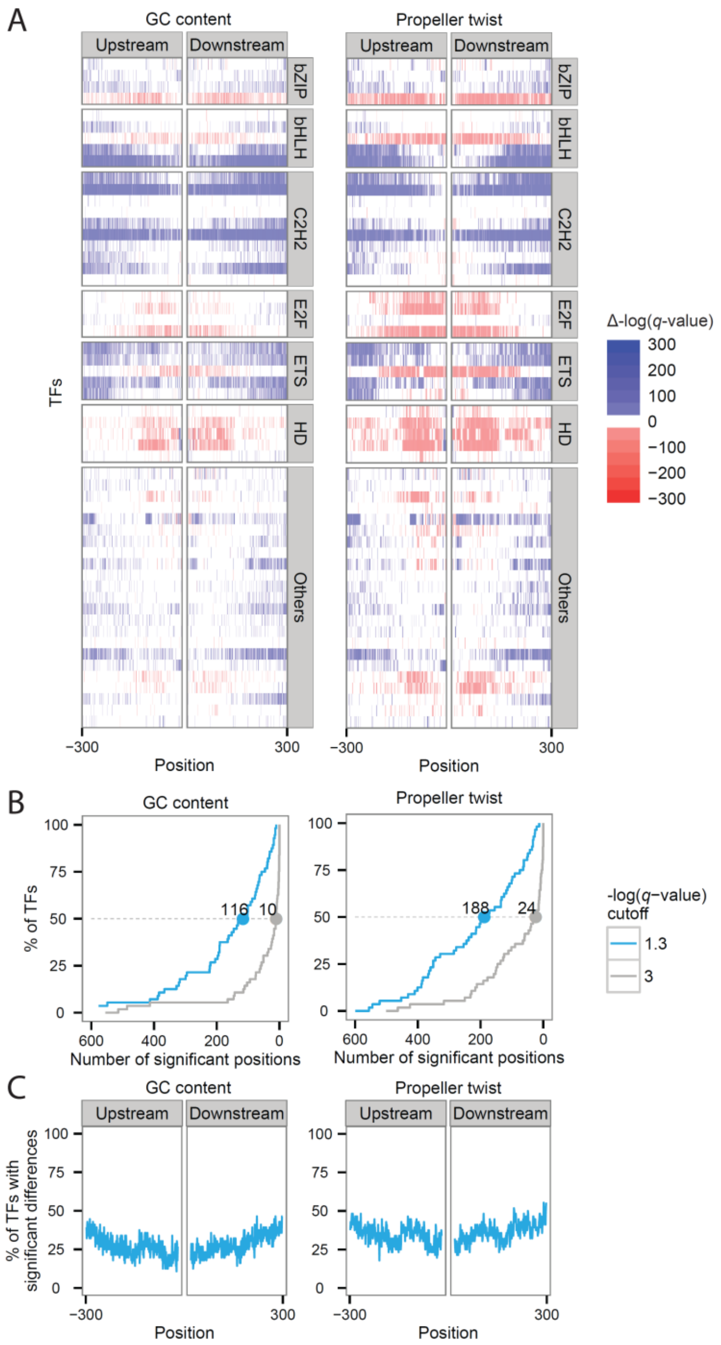

Supplemental Fig. 6: Differences in features of the regions surrounding TF motifs in a subset of bound and unbound sequences that have similar motif compositions extracted from *in vivo* data. (A) Heat map representing the differences in GC content (*left*), propeller twist (*right*) 300 bp up- and downstream of the core motifs, whereby red indicates positions at which the respective feature value was lower in the bound motifs, and blue represents positions at which the respective feature value was higher in the bound compared to the unbound motifs (the color intensity represents the significance).

Supplemental Fig. 7

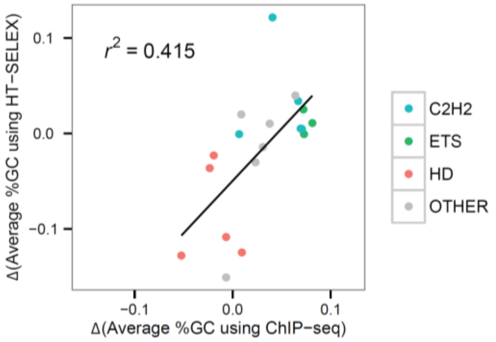

Supplemental Fig. 7: Comparison of *in vitro* and *in vivo* GC preferences. The scatter plot shows the difference in average GC content between the bound and the unbound sequences of TFs that are present in both data-sets. The TFs are colored according to the color code used for TF families: cyan for C2H2 TFs, green for ETS TFs, red for homedomains, and all others in grey; black line shows the linear regression trend line. The r^2 of the trend line is shown above.

Supplemental Fig. 8

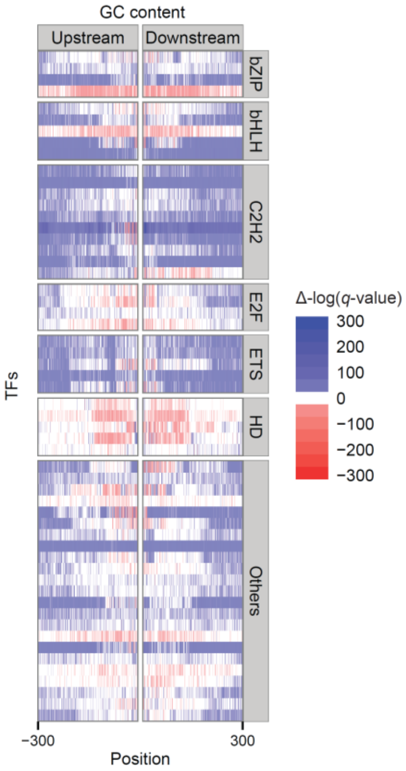

Supplemental Fig. 8: Differences in GC content of the regions surrounding TF motifs in bound and unbound sequences extracted from *in vivo* data where all peaks overlapping with the highest and lowest 10% GC content promoters were removed. Heat map representing the differences in GC content 300 bp up- and downstream to the core motifs, whereby red indicates positions at which the GC content was lower in the bound motifs, and blue represents positions at which the GC content was higher in the bound compared to the unbound motifs (the color intensity represents the significance). The TFs were grouped by the different TF families (FH for forkhead, HD for homeodomain). The positions correspond to the core-binding motif.

Supplemental Fig. 9

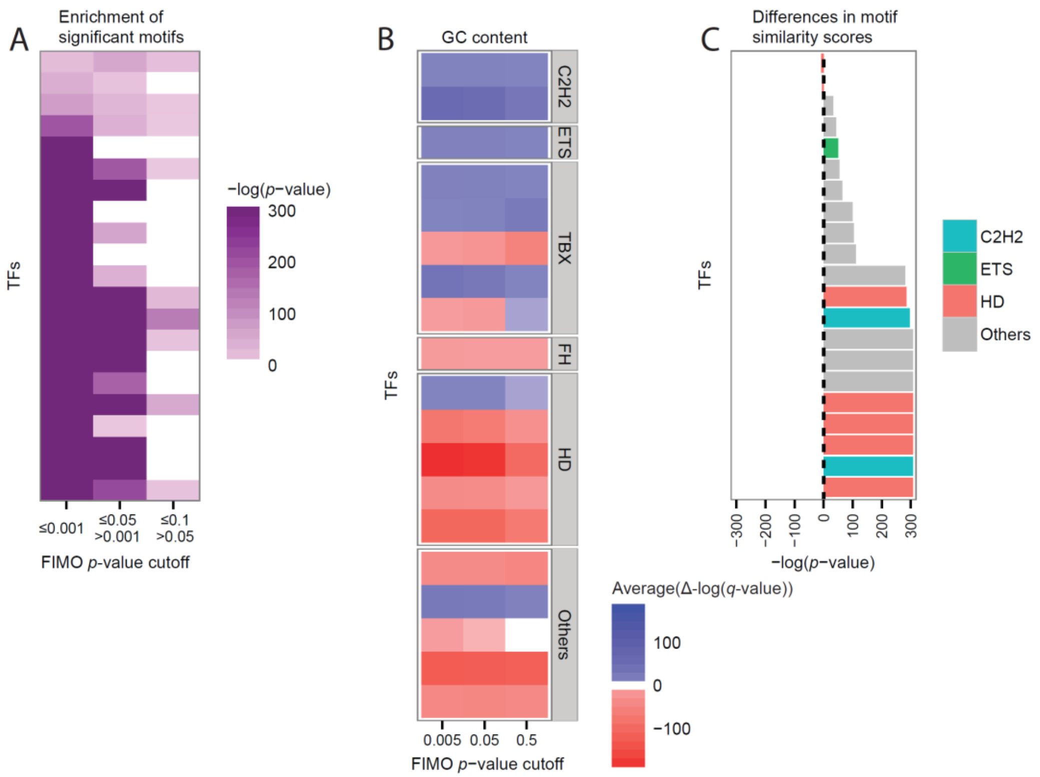

Supplemental Fig. 9: *In vitro* motif similarity differences. (A) Wilcoxon test *p*-values comparing the number of significant motifs found surrounding bound and unbound sequences, using different FIMO *p*-value cutoffs for defining significant motifs. Purple represents TFs for which their bound sequences had a significantly higher number of significant motifs than their unbound sequences. The color intensity represents the statistical significance. (B) Heat map representing the Wilcoxon test *p*-values of the differences in GC content 10 bp up- and downstream of the core motifs, where positions showing significant similarity to the PFM were removed (using different FIMO *p*-value cutoffs for defining significant motifs). Red indicates TFs for which the GC content was lower in the bound motifs, and blue represents TFs for which the GC content was higher

in the bound compared to the unbound motifs (the color intensity represents the statistical significance). The TFs were grouped by the different TF families (FH for forkhead, HD for homeodomain). (C) Comparison of the PFM similarity scores between sequences surrounding *in vitro* bound and unbound motifs. The bars on the right side represent TFs having higher motif similarity scores in the bound sequences, and bars on the left represent TFs having lower similarity scores in the bound sequences. The height of the bar represents the significance of the differences between the groups using Wilcoxon test. The broken line represents the significant cutoff using the shuffled data. The TFs are colored according to the color code used for TF families: cyan for C2H2 TFs, green for ETS TFs, red for homedomains, and all others in grey.

Supplemental Fig. 10

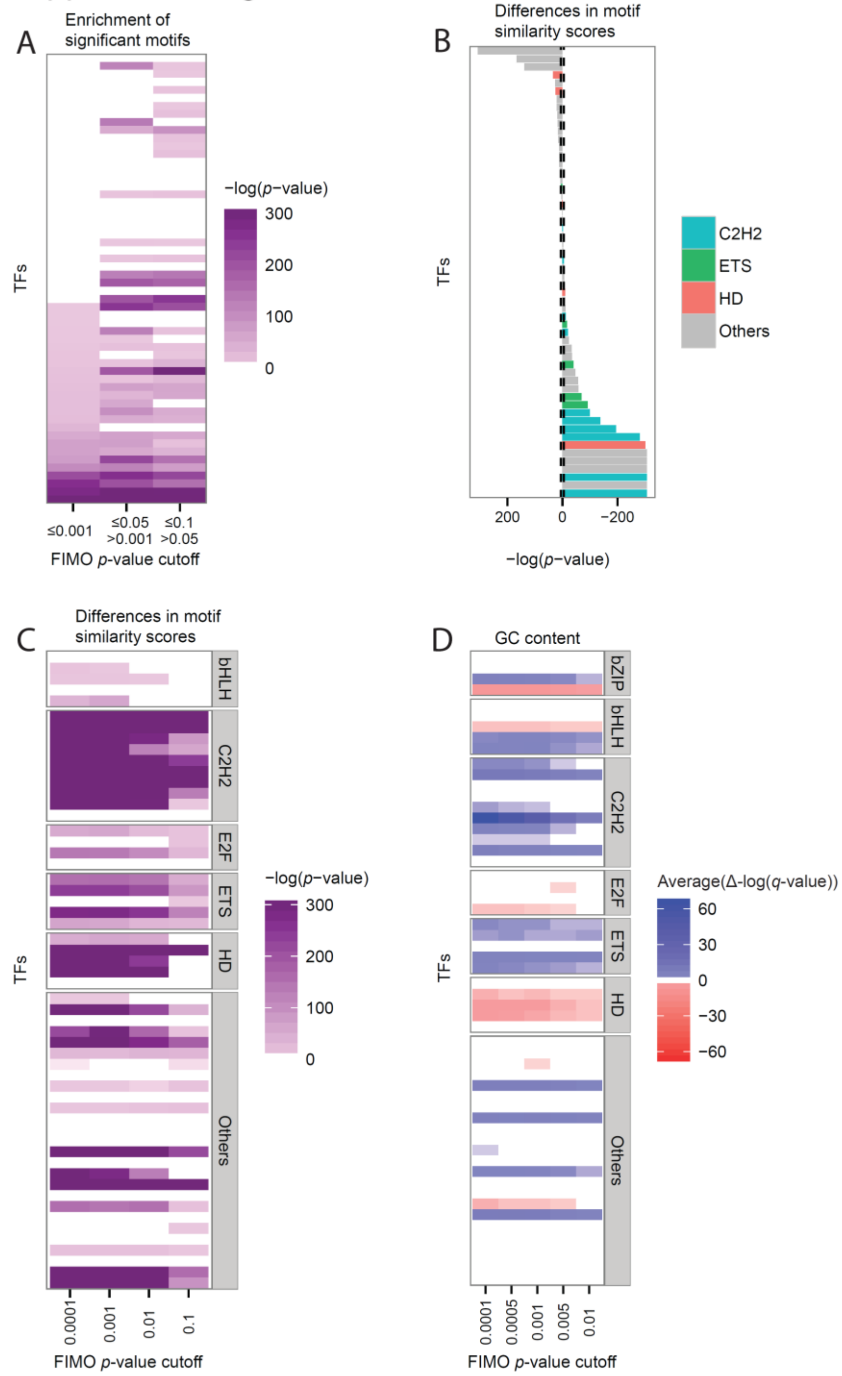

Supplemental Fig. 10: *In vivo* motif similarity differences. (A) Wilcoxon test *p*-values comparing the number of significant motifs found surrounding bound and unbound sequences, using different FIMO *p*-value cutoffs for defining significant motifs (the color intensity represents the statistical significance). (B) Comparison of the PFM similarity scores between sequences surrounding *in vivo* bound and unbound motifs, where sequences with FIMO *p*-value scores ≤0.001 (not including the core motif) were

removed. The bars on the right side represent TFs having higher motif similarity scores in the bound sequences, and bars on the left represent TFs having lower similarity scores in the bound sequences. The height of the bar represents the significance of the differences between the groups. The broken line represents the significant cutoff using the shuffled data. The TFs are colored according to the color code used for TF families: cyan for C2H2 TFs, green for ETS TFs, red for homedomains, and all others in grey. (C) Wilcoxon test *p*-values comparing PFM similarity scores between sequences surrounding *in vivo* bound and unbound motifs, where positions showing significant similarity to the PFM were removed (using different FIMO *p*-value cutoffs for defining significant motifs). Purple represents TFs for which the PFM similarity scores of their bound sequences were significantly higher than their unbound sequences. The color intensity represents the statistical significance. The TFs were grouped by the different TF families (FH for forkhead, HD for homeodomain). (D) Wilcoxon test *p*-values of the average differences in GC content 100 bp up- and downstream of the core motifs, where positions showing significant similarity to the PFM were removed (using different FIMO *p*-value cutoffs for defining significant motifs). Red indicates TFs for which the GC content was lower in the bound motifs, and blue represents TFs for which the GC content was higher in the bound compared to the unbound motifs (the color intensity represents the statistical significance). The TFs were grouped by the different TF families (FH for forkhead, HD for homeodomain).

Supplemental Fig. 11

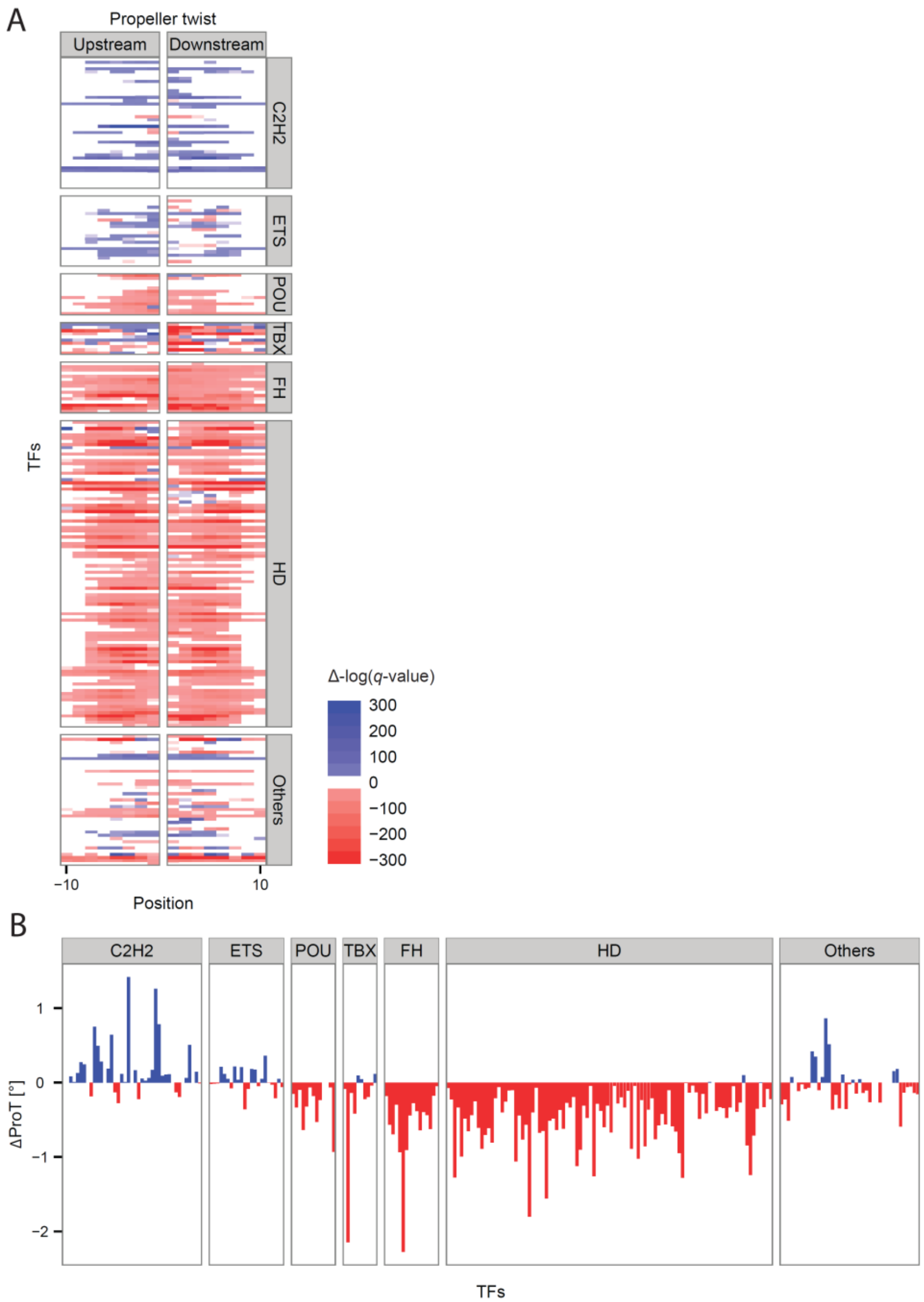

Supplemental Fig. 11: Differences in propeller twist in the regions surrounding TF motifs in bound and unbound sequences extracted from *in vitro* data. (A) Heat map representing the differences in propeller twist up- and downstream of the core motifs, whereby red indicates positions with enhanced negative propeller twist in the bound motifs, and blue represents positions with less pronounced propeller twist in the bound compared to the unbound motifs (the color intensity represents the statistical

significance). The TFs were grouped by the different TF families (FH for forkhead, HD for homeodomain). The positions correspond to the core-binding motif. (B) Differences between the average propeller twist of bound and the unbound sequences, red indicates TFs which prefer binding to regions with enhanced negative propeller twist, blue represents TFs which prefer binding to sequences with less pronounced propeller twist.

Supplemental Fig. 12

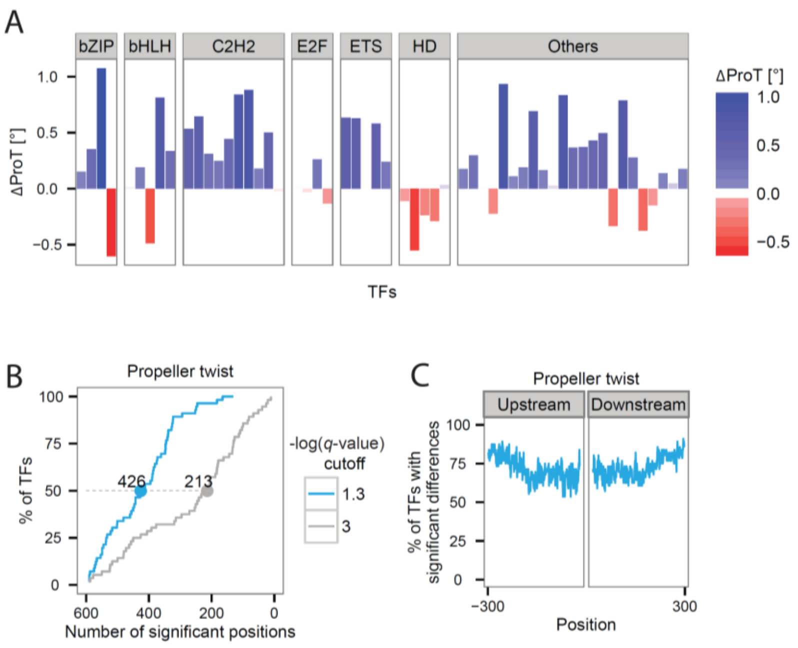

Supplemental Fig. 12: Differences in propeller twist in the regions surrounding TF motifs in bound and unbound sequences extracted from *in vivo* data. (A) Differences between the average propeller twist of bound and unbound sequences, whereby red indicates TFs which prefer binding to regions with enhanced negative propeller twist, blue represents TFs which prefer binding to sequences with less pronounced propeller twist. (B) Cumulative plot representing the proportion of TFs as a function of the number of surrounding positions that differ significantly between the bound and unbound groups using two different thresholds to define significant differences: -log(*q*-value)≥ 1.3 in blue, and ≥3 in grey. (C) Plot showing the percentage of TFs with significant differences (-log(*q*-value)≥ 1.3) for each position 300 bp up- and downstream of the core motif.

Supplemental Fig. 13

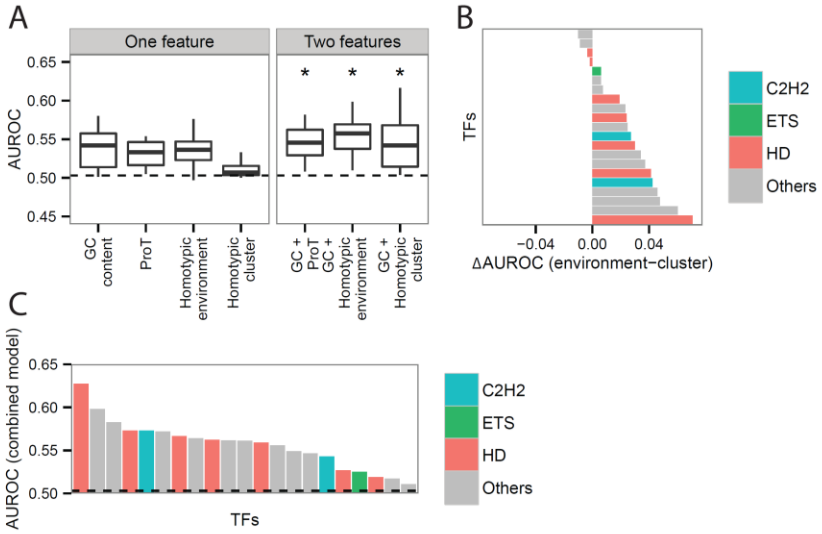

Supplemental Fig. 13: Predicting bound and unbound TF motifs *in vitro*. (A) L2-regularized multiple linear regression (MLR) models based on one or two features *in vitro*. The features characterizing the average GC content (GC content), propeller twist (ProT), the average PFM similarity scores (Homotypic environment), and the summary of all significant similarity scores (using a FIMO *p*-value cutoff of 0.001) (Homotypic cluster). All features were extracted up- and downstream of the core motif, excluding the core motif. The box plots represent the distribution of the area under the receiver operating characteristic (AUROC) for all 21 TFs using one or two features. The dashed line represents the maximum AUROC obtained using randomly shuffled data. Asterisks are shown for features in which the AUROC obtained using the two-feature model is significantly higher than the AUROC obtained using only GC content. (B) For each TF, comparison of the AUROC obtained using the Homotypic environment model and the Homotypic cluster model. (*C*) AUROC values for each of the 21 TFs separately employing a model that incorporates the best performing features: GC content, propeller twist, and homotypic environment. Dashed line represents the maximum AUROC obtained using randomly shuffled data. The TFs are colored according to the color code used for TF families: cyan for C2H2 TFs, green for ETS TFs, red for homedomains, and all others in grey.

Supplemental Fig. 14

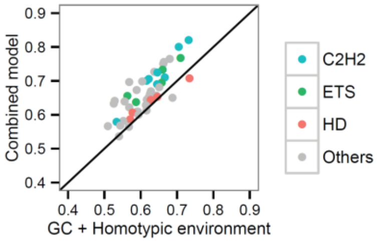

Supplemental Fig. 14: Comparison of *in vivo* multiple linear regression (MLR) AUROC of each TF using the GC + Homotypic environment features and the combined model. The TFs are colored according to the color code used for TF families: cyan for C2H2 TFs, green for ETS TFs, red for homedomains, and all others in grey.

Supplemental Fig. 15

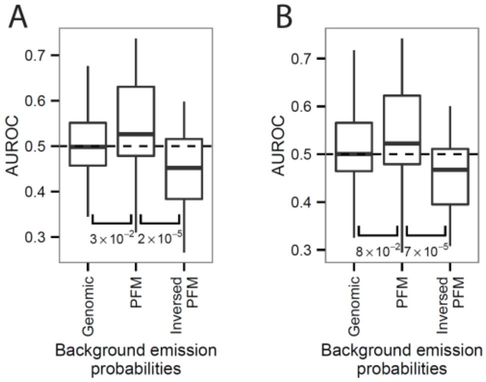

Supplemental Fig. 15: AUROC of the Hidden Markov Models (HMMs) where the transition probability of moving from the motif state to the background state was set to (A) 0.9 or (B) 0.8. Three different HMM models were implemented using different emission probabilities for the background state: the genomic nucleotide frequency, average nucleotide frequency of the PFM, and the inversed average nucleotide frequency of the PFM. Wilcoxon test *p*-values are shown below. The dashed line represents an AUROC of 0.5.

Supplemental Fig. 16

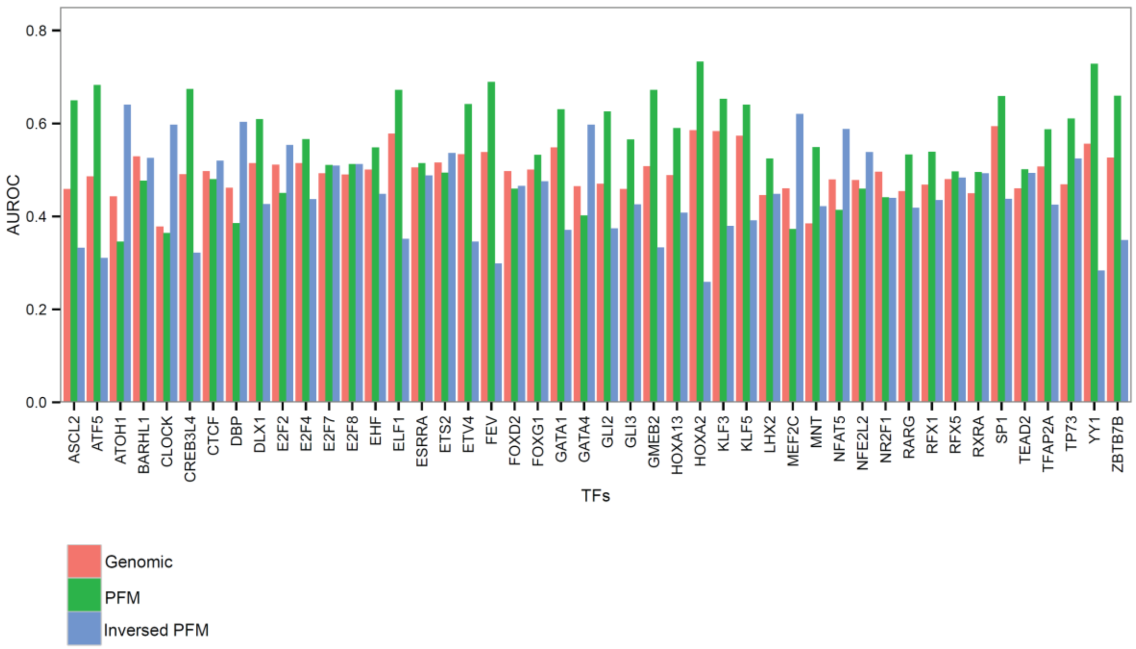

Supplemental Fig. 16: AUROC values for each of the TFs separately employing the three Hidden Markov Models (HMMs).

Supplemental Fig. 17

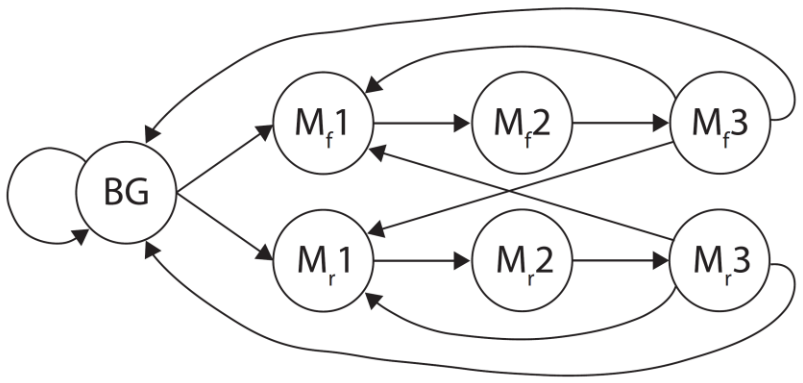

Supplemental Fig. 17: Hidden Markov Model (HMM) scheme. The first state represents the background and the following states represent the PFM on the forward strand (M_f) and the reverse strand (M_r).

Supplemental Fig. 18

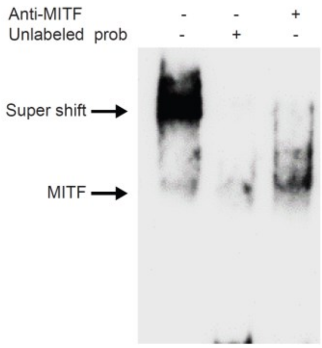

Supplemental Fig. 18: Supershift analyzed with probes corresponding to the MITF binding region of the *TRPM1* promoter. Highly expressing MITF melanoma cell (WM3682) nuclear extracts were used as a source of MITF. WT Biotinylated, WT unlabeled probe and polyclonal anti-MITF (MITF) antibody was used for the analyses. The MITF binding probe and super shifts are marked with arrows.
